# Uncovering the role of c-Fos in the bidirectional relationship between depression/anxiety behaviors and α-synuclein propagation in Parkinson’s disease

**DOI:** 10.1016/j.neurot.2025.e00807

**Published:** 2025-11-26

**Authors:** Soo-Jeong Kim, Jae-Bong Kim, Seonghui Ham, Sang Myun Park

**Affiliations:** a Center for Convergence Research of Neurological Disorders, Ajou University School of Medicine, Suwon, South Korea; b Department of Pharmacology, Ajou University School of Medicine, Suwon, South Korea; c Neuroscience Graduate Program, Department of Biomedical Sciences, Ajou University School of Medicine, Suwon, South Korea

**Keywords:** α-synuclein, Anxiety, Depression, Chronic restraint stress, c-Fos

## Abstract

Parkinson’s disease (PD) presents with both motor and non-motor symptoms, including depression and anxiety, which often precede motor onset, yet the mechanisms linking these affective symptoms to PD pathology remain unclear. In this study, we investigated the bidirectional relationship between depression/anxiety behaviors and α-synuclein (α-syn) propagation using A53T α-syn transgenic mice subjected to chronic restraint stress (CRS) and/or intrastriatal injection of α-syn preformed fibrils (PFFs). Behavioral testing and immunohistochemical analyses revealed that CRS enhanced PFF-induced α-syn propagation and exacerbated depression/anxiety-like behaviors, while α-syn propagation was associated with aggravated CRS-induced behavioral deficits, indicating a potential reciprocal association that could contribute to accelerating PD progression. This interaction was mediated by the neuronal activity marker c-Fos. Pharmacological inhibition of c-Fos with T5224 mitigated both behavioral and pathological changes, and mGluR5 activation was found to partially contribute to c-Fos induction and α-syn spread. Together, these findings highlight a feedback interaction between affective symptoms and α-syn pathology in PD, mediated in part by neuronal activity–dependent mechanisms involving c-Fos and mGluR5, and suggest that early interventions targeting both neuronal activity and α-syn propagation may slow PD progression and improve patient quality of life.

## Graphical abstract

**Figure:**
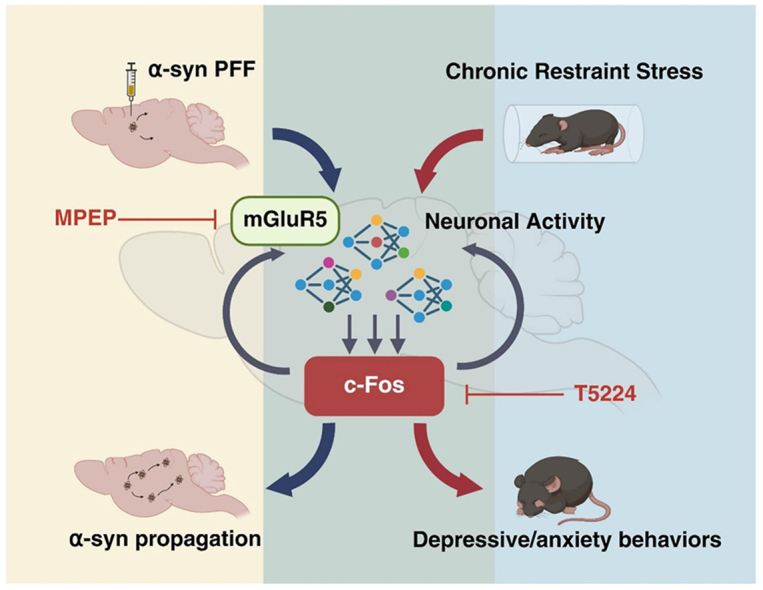
Schematic summary of how chronic stress and α-synuclein propagation interact via c-Fos–mediated neuronal activity.

## Introduction

Parkinson’s disease (PD) is a common neurodegenerative disorder characterized by progressive dopaminergic neuronal loss in the substantia nigra pars compacta and the deposition of α-synuclein (α-syn) aggregates, forming Lewy bodies (LBs) and Lewy neurites (LNs) [1]. While motor symptoms such as tremor, bradykinesia, and gait disturbance define PD, non-motor symptoms, including sensory, autonomic, sleep, and psychiatric disturbances, are now recognized as core features [2,3]. These symptoms often outweigh motor impairments in their impact on quality of life and are strongly linked to greater disease burden and reduced well-being [[4], [5], [6]].

Depression and anxiety are prominent prodromal features linked to greater PD severity, poorer treatment response, and reduced functional capacity [[7], [8], [9]]. Depression affects ∼35 ​% of patients, with major depressive disorder present in 17 ​%, and both conditions markedly impair quality of life. [[10], [11], [12], [13]]. These symptoms further hasten cognitive and motor decline [14] and increased mortality [15,16], although the underlying mechanisms remain unclear. Lewy pathology, driven by prion-like α-syn propagation, is thought to originate in the olfactory bulbs and dorsal motor nucleus of the vagus nerve spreading to interconnected brain regions and ultimately cortical areas involved in affective regulation and stress response, potentially linking non-motor symptoms to PD progression [[17], [18], [19], [20]].

Neuronal activity critically regulates extracellular α-syn release, accounting for ∼70 ​% of total secretion [21]. Chronic neuronal activation elevates insoluble and pSer129 α-syn levels in the striatum, contributing to PD pathogenesis [22]. Similar activity-dependent mechanisms mediate the release and spread of tau and amyloid-β in Alzheimer’s disease (AD) [23,24]. Stress robustly induces c-Fos expression across brain regions implicated in affective regulation, many of which overlap with α-syn pathology in PD [[25], [26], [27], [28], [29], [30], [31], [32]]. Elevated neuronal activity in specific regions such as the medial prefrontal cortex, ventral tegmental area, raphe nucleus, lateral habenula, amygdala, and paraventricular hypothalamic nucleus has been linked to depression/anxiety behaviors [[33], [34], [35], [36], [37], [38]].

We therefore hypothesize that chronic restraint stress (CRS)-induced depression and anxiety enhance activity-dependent extracellular α-syn release and propagation, while α-syn spread further exacerbates these affective symptoms, creating a pathogenic feedback interaction. To test this detrimental bidirectional relationship, we employed a combined preformed fibril (PFF) injection and CRS paradigm to investigate the interplay between mood disturbances, neuronal hyperactivity, and α-syn pathology in PD.

## Materials and methods

### Animals

M83 transgenic (Tg) mice overexpressing human A53T α-syn under the prion protein promoter (B6; C3-Tg(Prnp-SNCA∗A53T)83Vle/J, The Jackson Laboratory) and C57BL/6 mice (Orient Bio) were used [39]. Hemizygous M83 mice were obtained by crossing male homozygous M83 with female C57BL/6 mice. Animals were housed under a 12 h light/dark cycle with ad libitum food and water. Experiments were conducted between 09:00 and 18:00 ​h under standard lighting. All animal procedures were performed in compliance with the guidelines approved by the Ajou University School of Medicine Ethics Review Committee (IACUC No. 2022-0014).

### Stereotaxic surgery and drug treatment

Recombinant human WT α-syn and PFFs were prepared as described [39]. Hemizygous male M83 mice (2–3 months) were anesthetized with 2 ​% 2,2,2-tribromoethanol (250 ​mg/kg, i.p., Sigma-Aldrich, Germany) and placed in a stereotaxic frame. PBS or α-syn PFFs (10 ​μg in 2 ​μl; 400 ​nl/min) were unilaterally injected into the dorsal striatum (AP ​+ ​1.0 ​mm, ML ​+ ​1.8 ​mm, DV – 3.2 ​mm) using a 33-gauge needle, left in place for 10 ​min before withdrawal. Following injection, mice received 3-{5-[4-(cyclopentyloxy)-2-hydroxybenzoyl]-2-[(3-hydroxy-1,2-benzisoxazol-6-yl)methoxy]phenyl}propionic acid) (T5224, MedChemExpress, USA), starting at 3 days post-injection. T5224 was dissolved in 10 ​% DMSO and 90 ​% polyvinylpyrrolidone solution or corn oil. T5224 (30 ​mg/kg, p.o.) or vehicle was administered daily for 4 weeks and subsequently used for behavioral and immunohistochemical analyses. 2-Methyl-6-(2-phenylethynyl)-pyridine (MPEP monohydrochloride, Cayman Chemical, USA) was dissolved in 10 ​% Tween 80 (vol/vol) and 90 ​% sterile saline. MPEP (3 ​mg/kg) or vehicle, was administered intraperitoneally (i.p.) for 4 weeks and subsequently used for immunohistochemical analyses.

### Chronic restraint stress

Mice were randomly divided into the nonstressed control (NCRS) and stressed (CRS) groups. Chronic restraint stress (CRS) experiments were performed using acrylic cylindrical restrainers with large flat bottoms (Jeungdo Bio & Plant Co., Seoul, Korea) for 2 ​h per day (between 15:00 and 17:00 ​h) for 28 days in total. Each restrainer is equipped with multiple slots to accommodate mice of different sizes, effectively limiting limb movement without causing pain. After restraint, the mice were promptly returned to their home cages. Non-restrained mice remained in their home cages without undergoing the CRS procedure, while CRS-exposed mice were denied access to food and water during the restraint period. Mouse body weight was recorded weekly throughout the experiment.

### Open field test

Mice were placed in the testing room to allow habituation to the environment. They were then placed into a white acrylic 40 ​× ​40 ​× ​35 ​cm open field box and monitored for 10 ​min under 3–50 lux conditions. The distance moved and the time spent in the center zone were recorded, and each recorded video was analyzed using SMART 3.0 software (Panlab Harvard Apparatus, Barcelona, Spain).

### Sucrose preference test

Sucrose preference was assessed following CRS using a two-bottle choice test. Mice were habituated with two bottles of 1 ​% sucrose for 24 ​h, followed by 24 ​h with one bottle replaced by water. After 18 ​h of water deprivation, mice were presented with pre-weighed bottles containing 2 ​% sucrose and water for 12 ​h. Consumption was measured by weight difference, and sucrose preference was calculated as the ratio of sucrose intake to total fluid intake.

### Elevated plus-maze test

The elevated plus maze consisted of two open arms and two closed arms (30 ​× ​6 ​cm, ∼20 lux), along with a central zone, elevated 50 ​cm above the floor. Mice were initially positioned in the central zone facing a closed arm and allowed to freely explore the maze for 5 ​min. The number of entries into the open and closed arms, as well as the time spent in each arm, were recorded and analyzed using SMART 3.0 software.

### Light-dark transition test

The apparatus consisted of two compartments, a dimly lit chamber (∼2 lux) and a brightly illuminated chamber (390 lux), separated by a partition with an opening. Mice were allowed to freely explore both compartments for 10 ​min. The number of entries and time spent in the illuminated chamber were recorded and analyzed using SMART 3.0 software.

### Forced swim test

5-liter glass beakers were filled with 3–3.5 ​L of tap water at 25 ​°C. Mice were exposed to the water for 15 ​min on day one and 5 ​min on day two. Immobility (floating) and active swimming behaviors were quantified using SMART 3.0 software.

### Pole-climbing test

The pole-climbing test was performed using a 50 ​cm-high, 1 ​cm-diameter wooden pole wrapped in gauze. Bedding was placed at the base for safety. Mice were placed ∼7.5 ​cm below the top, facing upward, and trained in three sessions before testing. Latency to descend was measured (cutoff: 120 ​s) and recorded as turn time and total time.

### Grip strength test

Neuromuscular strength was evaluated by measuring the maximum forelimb grip force using a force transducer (Bioseb, USA). Mice grasped a metal grid while gentle tail traction was applied, and the peak force at release was recorded digitally in grams.

### Rotarod test

Motor coordination and balance were assessed using a Rotarod apparatus (Bioseb, USA). Mice were placed on individual lanes with an empty lane between each to prevent interference. The rotarod accelerated from 4 to 40 ​rpm over 300 ​s. Each mouse performed three trials with at least 30 ​min rest between trials. The latency to fall was recorded as the measure of motor performance.

### Footprint test

Footprints were obtained by dipping the front and hind limbs of mice into non-toxic paint and allowing them to walk along a runway leading to an enclosed box. The runway floor was lined with white paper to capture the footprints. Stride length was analyzed by measuring the average distance between the front and hind limb prints. Data from mice that did not traverse the runway smoothly were excluded from the analysis.

### Immunohistochemistry

Brains were perfused with PBS, post-fixed in 4 ​% paraformaldehyde overnight, cryoprotected in 30 ​% sucrose, and coronally sectioned at 35 ​μm (Leica CM-3050). Sections (≥3/mouse) were permeabilized in 1 ​% Triton X-100/5 ​% BSA (PBS) for 60 ​min, then incubated at 4 ​°C for 8–12 ​h with primary antibodies: anti-c-Fos (CST #2250, 1:500), anti-pSer129 α-syn (Abcam #ab51253 or Wako #015-25191, 1:500), anti-calbindin (Swant #CB300, 1:1000), and anti-CaMKII (Abcam #ab22609, 1:500) in blocking solution. Sections were washed in PBS and incubated for 1 ​h at room temperature with Alexa Fluor 488– or 568–conjugated secondary antibodies (Invitrogen; 1:500). After PBS washes, sections were mounted on silane-coated glass slides (Muto Glass) using Vectashield (Vector). Immunofluorescence was imaged with a Zeiss LSM800 or Leica TCS SP8 confocal microscope, and quantification was performed using MetaMorph software (Molecular Devices). Free-floating sections were rinsed in PBS, incubated in 3 ​% H_2_O_2_/PBS for 5 ​min, and blocked in 1 ​% BSA/0.2 ​% Triton X-100 (PBS) for 1 ​h. Primary antibodies—anti-pSer129 α-syn (Wako #015-25191, 1:5000), anti-Iba-1 (Wako #019-19741, 1:1000), anti-GFAP (Neuromics #RA22101, 1:5000), and anti-TH (Abcam #ab13772, 1:1000)—were applied overnight at 4 ​°C. Sections were incubated with biotinylated secondary antibodies (Vector) for 1 ​h, followed by ABC reagent (Vector) for 1 ​h, and visualized with DAB (Sigma-Aldrich). After mounting on slides, tissues were dehydrated, cleared, and coverslipped with Permount.

### Fluorescent in situ *hybridization (RNAscope assay)*

Fresh-frozen tissue sections (14 ​μm) were mounted on Superfrost Plus slides (Fisher Scientific) and stored at −80 ​°C until RNAscope ISH. Probes for mGluR5 (#1108071, targeting bp 1199–2142 of Grm5 mRNA) and c-Fos (#316921-C2, targeting bp 407–1427 of FOS mRNA) were obtained from Advanced Cell Diagnostics. ISH was performed according to the manufacturer’s protocol. Slides were coverslipped with ProLong Gold antifade medium (Invitrogen) and imaged at 40 ​× ​with a Zeiss LSM800 confocal microscope.

### Quantitative pathology

Slides were scanned at 20 ​× ​magnification using an Axio Scan.Z1 slide scanner (Carl Zeiss) with consistent imaging parameters. Z-stack images were converted to maximum-intensity projections and processed in ZEN software. For α-syn pathology quantification, coronal sections at bregma +2.10, +0.98, −1.58, and −3.08 ​mm were analyzed in MetaMorph (Molecular Devices) to measure the percentage area of pSer129 α-syn and c-Fos immunoreactivity. Annotations were standardized for each brain region according to Paxinos and Franklin’s Mouse Brain Atlas. IBA-1 density was quantified by counting IBA-1-positive cells at 20 ​× ​magnification (Zen Blue 2.3), expressed as cells/mm^2^. GFAP and striatal TH fiber intensities were measured from 5 to 6 fields per region in three sections/mouse, with background values subtracted and results normalized to percentages using ImageJ. TH-positive neuron counts followed established protocols [40]. Briefly, TH-positive neurons in the substantia nigra (SN) were quantified from 5 to 6 free-floating sections (35 ​μm, 1:6 series), with counts multiplied by six to estimate total cell numbers per mouse.

### Quantification and statistical analysis

Statistical analyses were performed using GraphPad Prism 8. Normally distributed data were analyzed by Student’s t-test for pSer129 α-syn area comparisons between CRS and NCRS groups, and by two-way ANOVA for CRS/NCRS ​× ​PFF/PBS conditions, followed by Tukey’s post hoc tests. Pearson’s correlation coefficients were calculated where applicable. Data are presented as mean ​± ​SEM, with significance denoted as ∗∗∗*P* ​< ​0.001, ∗∗*P* ​< ​0.01, ∗*P* ​< ​0.05.

## Results

### Injection of PFF accelerates depression/anxiety-like behaviors induced by CRS

**Fig. 1:**
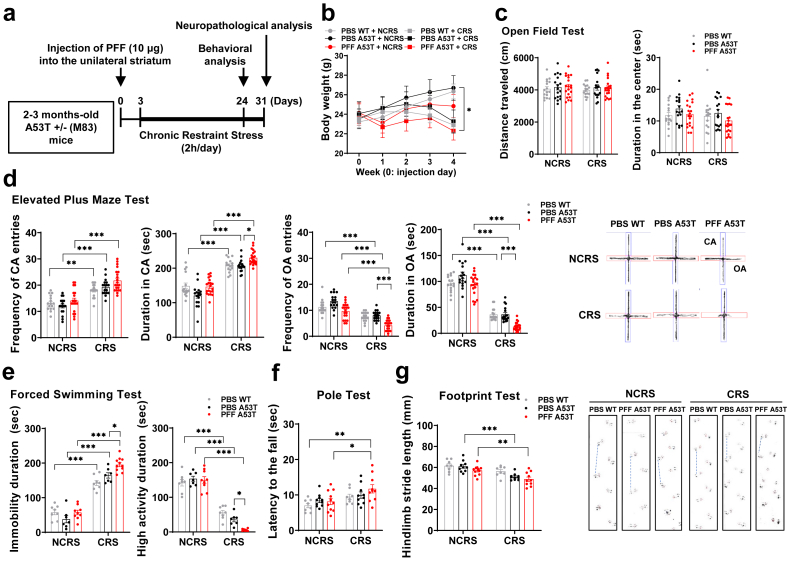
**PFF injection exacerbates anxiety- and depression-like behaviors under CRS exposure in A53T α-syn Tg mice.** (a) Schematic diagram of the experimental procedure. (b) The monitoring of body weight over the 4 weeks of CRS, with the corresponding time course shown. (c) Locomotor activity was assessed by measuring the total distance traveled and duration in the center area of the open field. (d) Anxiety-like behaviors were measured by time spent in the closed and open arms, as well as the frequency of entries into the closed and open arms (CA and OA) of the elevated plus maze. Representative traces showing movements in the elevated plus maze are presented. (e) Depression-like behaviors were measured by immobility and high activity time in the forced swimming test. (f) Motor function was evaluated by the latency to climb down in the pole test. (g) The hindlimb stride lengths (cm) recorded in footprint analysis, with dotted lines representing the direction of walking progression, are shown. *n* ​= ​16–21 per group for a–d, *n* ​= ​8–11 per group for e–g. ∗∗∗*P* ​< ​0.001, ∗∗*P* ​< ​0.01, ∗*P* ​< ​0.05. One-way ANOVAs were performed for b, and Two-way ANOVAs were applied to c, d, e, f, and g with Tukey’s multiple comparison tests.

CRS, a standard paradigm for inducing depressive-like behaviors in rodents [41], was employed alongside the PFF injection model to investigate the bidirectional relationship between affective behaviors and α-syn propagation in A53T α-syn Tg and control mice (Fig. 1a). After three weeks, body weight did not differ between CRS and non-CRS groups; however, PFF-injected A53T α-syn Tg mice under CRS exhibited reduced weight gain compared with PBS-treated NCRS controls, by week four (Fig. 1b). CRS alone reduced open-arm exploration in the elevated plus maze (EPM) and increased forced swimming test (FST) immobility, with both effects further exacerbated by PFF injection, particularly in A53T α-syn Tg mice. PFF/CRS-treated A53T α-syn Tg mice exhibited the most pronounced anxiety- and depressive-like phenotypes (Fig. 1d and e), while locomotor activity in the open field test (OFT) remained unchanged (Fig. 1c). In the light/dark box (LDT) and sucrose preference (SPT) tests, PFF-injected mice under CRS showed no significant differences (Supplementary Fig. 1a and b). Motor function was largely preserved, though PFF/CRS-treated A53T α-syn Tg mice displayed prolonged descent times in the pole test (Fig. 1f) and stride length alterations in footprint test (Fig. 1g), without changes in rotarod or grip strength performance (Supplementary Fig. 1c and Supplementary Fig. 1d). Overall, PFF injection potentiated depressive/anxiety-like behaviors in the EPM and FST, supporting a link between α-syn propagation and the exacerbation of affective symptoms.

### CRS accelerates α-syn pathology induced by PFF injection

**Fig. 2:**
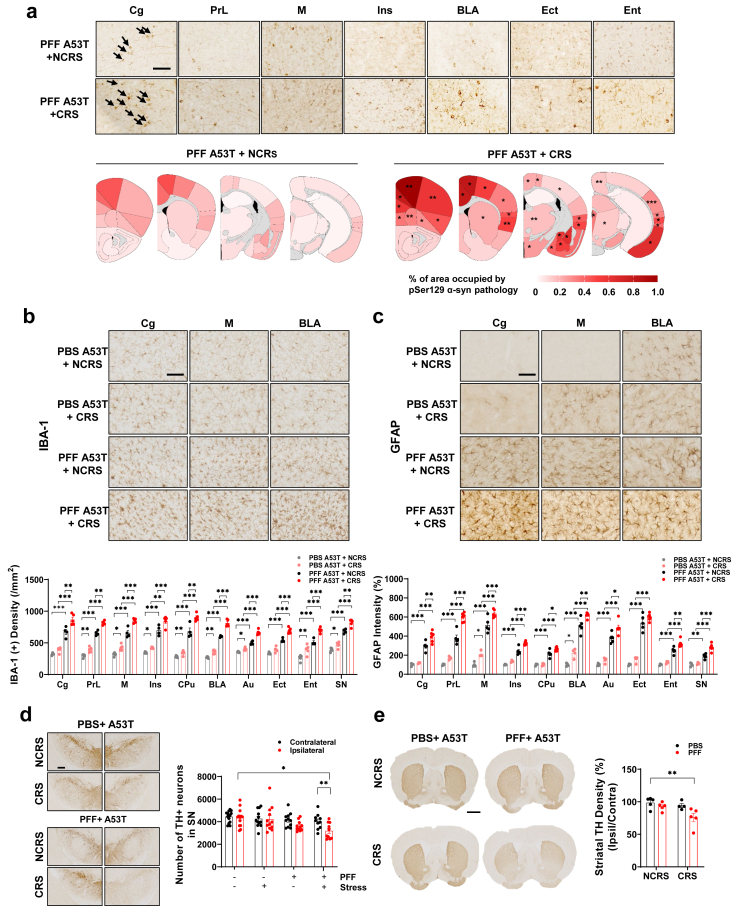
**CRS aggravates α-syn propagation and dopaminergic marker loss induced by PFF injection.** (a) Representative pSer129 α-syn pathology images are shown in Cg, PrL, M, Ins, BLA, Ect, and Ent of PFF ​+ ​NCRS or PFF ​+ ​CRS brain. Black arrows highlight pSer129 α-syn positive inclusion bodies. Scale bar, 50 ​μm. Percentage area occupied with α-syn pathology in ipsilateral regions. Heatmaps are representative of A53Tg mice analyzed with PFF ​+ ​NCRS (*n* ​= ​12), and PFF ​+ ​CRS (*n* ​= ​11) groups at 31 days post-injection. ∗∗∗*P* ​< ​0.001, ∗∗*P* ​< ​0.01, ∗*P* ​< ​0.05 compared with PFF ​+ ​NCRS, unpaired *t*-test, two-tailed. (b) Immunohistochemistry of IBA-1. IBA-1 density in ipsilateral Cg, PrL, M, Ins, CPu, BLA, Au, Ect, Ent, and SN was analyzed. Bar graphs summarizing quantitative results presented with PBS ​+ ​NCRS (*n* ​= ​5), PBS ​+ ​CRS (*n* ​= ​5), PFF ​+ ​NCRS (*n* ​= ​4) and PFF ​+ ​CRS (*n* ​= ​5) groups in microgliosis. (c) Immunohistochemistry of GFAP. GFAP intensities in ipsilateral Cg, PrL, M, Ins, CPu, BLA, Ect, Ent, and SN were analyzed. Bar graphs summarizing quantitative results presented with PBS ​+ ​NCRS (*n* ​= ​4), PBS ​+ ​CRS (*n* ​= ​4), PFF ​+ ​NCRS (*n* ​= ​5), and PFF ​+ ​CRS (*n* ​= ​6) in astrogliosis. ∗∗∗*P* ​< ​0.001, ∗∗*P* ​< ​0.01, ∗*P* ​< ​0.05, two-way ANOVA with Tukey’s multiple comparison test. Scale bar, 50 ​μm. (d) Immunohistochemistry of TH in the SN and analysis of stereology counts of TH-positive neurons in PBS ​+ ​NCRS (*n* ​= ​14), PBS ​+ ​CRS (*n* ​= ​12), PFF ​+ ​NCRS (*n* ​= ​11), or PFF ​+ ​CRS (*n* ​= ​10) groups. Scale bar indicates 200 ​μm. (e) Immunohistochemistry of TH in CPu and analysis of striatal TH density in PBS ​+ ​NCRS (*n* ​= ​5), PBS ​+ ​CRS (*n* ​= ​4), PFF ​+ ​NCRS (*n* ​= ​5) and PFF ​+ ​CRS (*n* ​= ​5) groups. ∗∗∗*P* ​< ​0.001, ∗∗*P* ​< ​0.01, ∗*P* ​< ​0.05, one-way and two-way ANOVA with, Tukey’s multiple comparison test. Scale bar indicates 1 ​mm. See Supplementary Fig. 2 for abbreviations used.

Immunohistochemical analysis of pSer129 α-syn, a marker of Lewy pathology, revealed widespread inclusions in A53T α-syn Tg mice one month after striatal PFF injection. CRS further enhanced their distribution, notably in the cingulate, prelimbic, motor, insular, ectorhinal, and entorhinal cortices, as well as the basolateral amygdaloid nucleus (Fig. 2a, Supplementary Fig. 2 and 3a, and Supplementary Table 1). These results demonstrate that PFF injection induces robust α-syn pathology, which is exacerbated by CRS in specific brain regions. Additionally, the association between α-syn pathology and neuroinflammation is well documented in mouse models of PD [40,42]. CRS also induced marked microgliosis and astrogliosis [43,44], as evidenced by increased Iba-1 (Fig. 2b and Supplementary Fig. 3b) and GFAP immunoreactivity (Fig. 2c and Supplementary Fig. 3c), which were further elevated in PFF-injected A53T α-syn Tg mice. Prior research has suggested that depression accelerates the degeneration of dopaminergic neurons [45,46]. However, within one month, PFF injection alone does not induce dopaminergic neuron degeneration [47]. While neither CRS nor PFF alone affected striatal TH intensity or substantia nigra TH-positive cell counts, combined CRS/PFF treatment reduced both measures, indicating exacerbated nigrostriatal dopaminergic neuron loss (Fig. 2d and e). These findings suggest that CRS accelerates α-syn seeding and spread, promoting neuroinflammation and progressive dopaminergic degeneration. Additionally, fluoro-Jade C (FJC) staining revealed increased neuronal degeneration in the substantia nigra of PFF-injected mice, which was further elevated by CRS (Supplementary Fig. 3d). These results support the notion that CRS-driven enhancement of α-syn seeding and propagation accelerates nigrostriatal dopaminergic neurodegeneration.

### c-Fos induction correlates with enhanced α-syn propagation induced by CRS

**Fig. 3:**
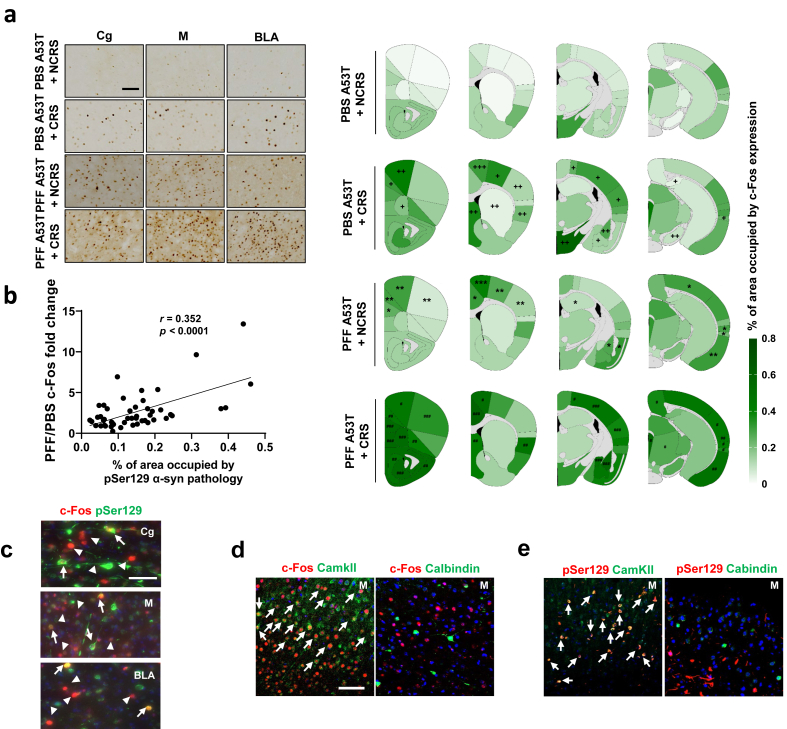
**CRS leads to increased α-syn propagation associated with c-Fos induction.** (a) Assessment of percentage area occupied by c-Fos in ipsilateral regions of PBS ​+ ​NCRS or PBS ​+ ​CRS, PFF ​+ ​NCRS or PFF ​+ ​CRS brain. Heatmap depicting quantitative results presented with PBS ​+ ​NCRS (*n* ​= ​3), PBS ​+ ​CRS (*n* ​= ​5), PFF ​+ ​NCRS (*n* ​= ​5) and PFF ​+ ​CRS (*n* ​= ​7) groups. +++ *P* ​< ​0.001, ++ *P* ​< ​0.01, + *P* ​< ​0.05 compared to PBS A53T ​+ ​NCRS, ∗∗∗*P* ​< ​0.001, ∗∗*P* ​< ​0.01, ∗*P* ​< ​0.05 compared to PBS A53T ​+ ​NCRS, and ###*P* ​< ​0.001, ##*P* ​< ​0.01, #*P* ​< ​0.05 compared to PFF A53T ​+ ​NCRS, unpaired *t*-test, two-tailed. (b) Correlation curve illustrating the relationship between the PFF/PBS c-Fos percentage area fold change and percentage area occupied by pSer129 α-syn. ∗*P* ​< ​0.001. (c) Representative images depict c-Fos (red, white arrowheads) and colocalization with pSer129 α-syn (white arrow) in Cg in the brain sections of mice injected with PFF. Scale bar, 50 ​μm. (d) Representative confocal images of excitatory and inhibitory markers (green; c-Fos used for costaining with CamkII and Calbindin) in M in the brain sections of mice injected with PFF. Scale bar, 50 ​μm. (e) Representative confocal images of excitatory and inhibitory markers (green; pSer129 α-syn used for costaining with CamkII and Calbindin) in M in the brain sections of mice injected with PFF. Scale bar, 50 ​μm. See also Supplementary Fig. 4.

To investigate whether altered neural activity contributes to α-syn pathology and behavioral deficits in PFF/CRS-treated A53T α-syn Tg mice, c-Fos expression was assessed as a marker of neuronal activation [48]. CRS induced c-Fos expression in various brain regions such as cingulate cortex, motor cortex, ventral orbital cortex, somatosensory cortex, insular cortex, septal, basolateral amygdaloid nucleus, basomedial amygdaloid nucleus, hypothalamus, parietal association cortex, perirhinal cortex, subiculum, and substantia nigra (Fig. 3a), aligning with findings from previous studies [[25], [26], [27], [28], [29], [30], [31], [32]]. Notably, c-Fos induction was more pronounced in the PFF/CRS groups (Fig. 3a and Supplementary Fig. 4a), suggesting a potential link between depression/anxiety-like behaviors and α-syn propagation. Intriguingly, PFF injection alone induced c-Fos expression in multiple regions, including the cingulate cortex, prelimbic cortex, motor cortex, somatosensory cortex, hippocampus, basolateral amygdaloid nucleus, visual cortex, ectorhinal cortex, perirhinal cortex, and entorhinal cortex. (Fig. 3a, Supplementary Fig. 4a, and Supplementary Table 2). The extent of c-Fos induction positively correlated with pSer129 α-syn levels (Fig. 3b), and a subset of c-Fos-positive neurons colocalized with pSer129 α-syn (Fig. 3c and Supplementary Fig. 4b). These findings suggest that CRS and PFF injection synergistically promote neuronal hyperactivity, potentially linking network dysfunction to α-syn propagation and affective-like behaviors.

### c-Fos-positive neurons are located adjacent to neurons bearing α-syn aggregates, as well as to excitatory neurons

Double-labeling revealed that while some c-Fos-positive neuronal somata colocalized with pSer129 α-syn, the majority did not, instead being located adjacent to α-syn-bearing neurons (Fig. 3c and Supplementary Fig. 4b). To determine whether this lack of colocalization was associated with different neuronal types, we performed co-staining of samples with antibodies against c-Fos or pSer129 α-syn and either CamKII, a marker for excitatory neurons, or Calbindin, a marker for inhibitory neurons. As shown in Fig. 3D and Supplementary Fig. 4c, most c-Fos were CamKII-positive, indicating a predominance of excitatory phenotype. Similarly, most pSer129-positive neurons were co-stained with CamKII but not Calbindin, suggesting that pSer129-positive neurons are also primarily excitatory (Fig. 3e and Supplementary Fig. 4d). These results imply that the neurons adjacent to those bearing α-syn inclusions are c-Fos-positive and are predominantly excitatory neurons. These findings suggest that c-Fos-positive excitatory neurons are positioned adjacent to α-syn inclusion bearing excitatory neurons.

### c-Fos inhibitor attenuates behavioral deficits related to non-motor symptoms and α-syn propagation induced by PFF injection or CRS exposure

**Fig. 4:**
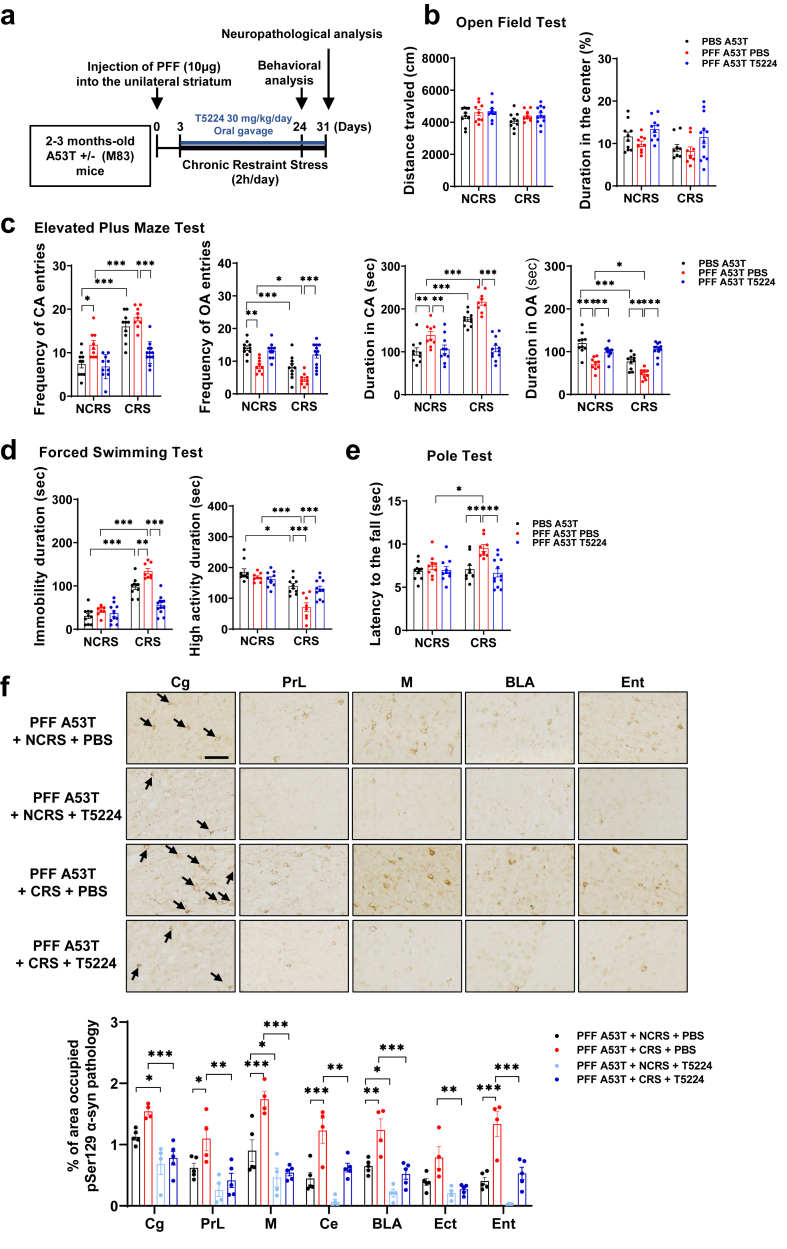
**c-Fos inhibitor attenuates behavioral deficits related to non-motor symptoms and α-syn propagation induced by PFF injection or CRS exposure.** (a) Schematic diagram of experiments for T5224 effects. The mice were treated with T5224 at 30 ​mg/kg/day by oral gavage (PO) administration for 4 weeks. (b) Locomotor activity across all groups was assessed using open-field tests, measured by the distance traveled and the time spent in the central region of the arena. One-way ANOVA with Tukey’s multiple comparisons. (c) Rescue of PFF-induced behavioral changes in the elevated plus maze by T5224 treatment. Two-way ANOVA with Tukey’s multiple comparisons, ∗∗∗*P* ​< ​0.001, ∗∗*P* ​< ​0.01, ∗*P* ​< ​0.05. PBS ​+ ​NCRS (*n* ​= ​10), PBS ​+ ​CRS (*n* ​= ​10), PFF ​+ ​NCRS ​+ ​PBS (*n* ​= ​9), PFF ​+ ​CRS ​+ ​PBS (*n* ​= ​9), PFF ​+ ​NCRS ​+ ​T5224 (*n* ​= ​10) and PFF ​+ ​CRS ​+ ​T5224 (*n* ​= ​12). (d) The reduced immobility time in the forced swim test was exhibited by mice treated with T5224. Two-way ANOVA with Tukey’s multiple comparisons, ∗∗∗*P* ​< ​0.001, ∗∗*P* ​< ​0.01, ∗*P* ​< ​0.05. PBS ​+ ​NCRS (*n* ​= ​10), PBS ​+ ​CRS (*n* ​= ​10), PFF ​+ ​NCRS ​+ ​PBS (*n* ​= ​8), PFF ​+ ​CRS ​+ ​PBS (*n* ​= ​8), PFF ​+ ​NCRS ​+ ​T5224 (*n* ​= ​10) and PFF ​+ ​CRS ​+ ​T5224 (*n* ​= ​12). (e) Rescue of PFF-induced behavioral changes in the pole test by T5224 treatment. Two-way ANOVA with Tukey’s multiple comparisons, ∗∗∗*P* ​< ​0.001, ∗∗*P* ​< ​0.01, ∗*P* ​< ​0.05. PBS ​+ ​NCRS (*n* ​= ​10), PBS ​+ ​CRS (*n* ​= ​10), PFF ​+ ​NCRS ​+ ​PBS (*n* ​= ​9), PFF ​+ ​CRS ​+ ​PBS (*n* ​= ​9), PFF ​+ ​NCRS ​+ ​T5224 (*n* ​= ​10) and PFF ​+ ​CRS ​+ ​T5224 (*n* ​= ​12). (f) T5224 treatment reverses the effects of PFF or CRS on α-syn pathology. Immunohistochemistry of pSer129 α-syn in ipsilateral Cg, PrL, M, Ins, CPu, BLA, Ect, and Ent was analyzed. Bar graphs depicting quantitative results presented with PFF ​+ ​NCRS ​+ ​PBS (*n* ​= ​5), PFF ​+ ​CRS ​+ ​PBS (*n* ​= ​4), PFF ​+ ​NCRS ​+ ​T5224 (*n* ​= ​4) and PFF ​+ ​CRS ​+ ​T5224 (*n* ​= ​5). Black arrows indicate pSer129 α-syn-positive inclusion bodies. Scale bar, 50 ​μm. Two-way ANOVA with Tukey’s multiple comparisons, ∗∗∗*P* ​< ​0.001, ∗∗*P* ​< ​0.01, ∗*P* ​< ​0.05. See also Supplementary Fig. 5a.

To investigate how c-Fos activation contributes to the reciprocal association between depression/anxiety behaviors and α-syn propagation, the AP-1–targeted c-Fos inhibitor T5224 was administered to PFF- and/or CRS-treated A53T α-syn Tg mice [49,50] (Fig. 4a). T5224 did not alter general locomotor activity in the OFT (Fig. 4b) but reversed anxiety- and depressive-like behaviors in the EPM and FST (Fig. 4c and d), as well as motor deficits in the pole test (Fig. 4e). Immunohistochemistry further showed that T5224 attenuated CRS-enhanced α-syn propagation (Fig. 4f and Supplementary Fig. 5a) and reduced pathology induced by PFF injection alone (Supplementary Fig. 5b), suggesting that c-Fos induction contributes to both affective disturbances and α-syn spread, and may represent a therapeutic target.

### mGluR5 mediates c-Fos induction by PFF, further α-syn propagation

**Fig. 5:**
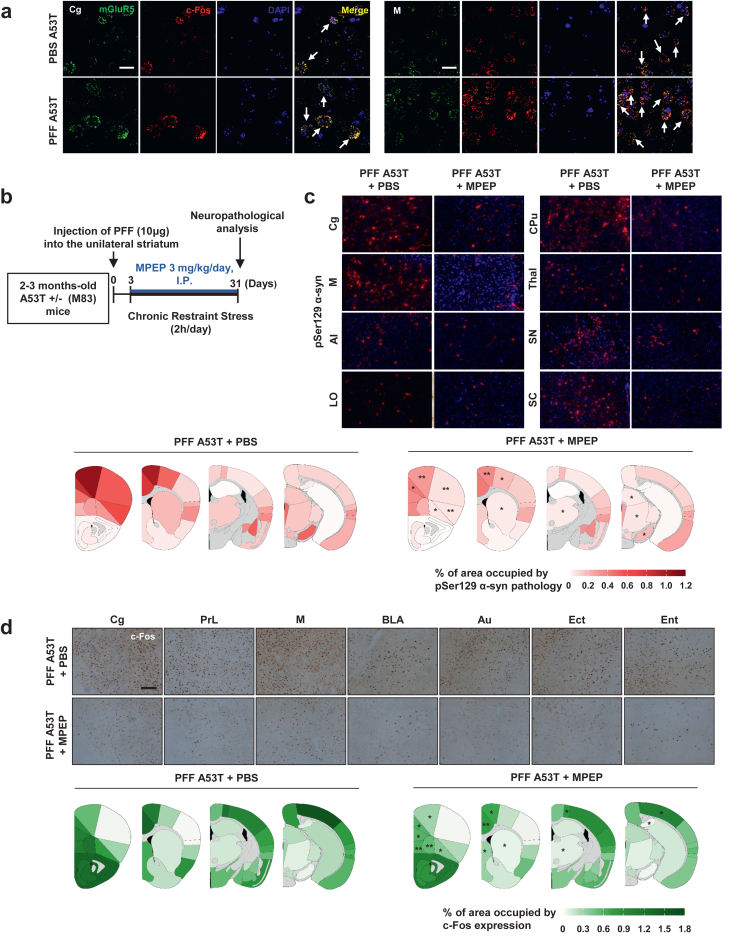
**mGluR5 mediates c-Fos induction associated with α-syn propagation.** (a) mGluR5 mRNA colocalizes with c-Fos-positive cells in Cg (arrows). Images illustrating c-Fos (red) and mGluR5 (green) mRNA colocalization (marked by white arrows) in Cg of A53T ​+ ​PBS and A53T ​+ ​PFF mice. Scale bar, 20 ​μm. (b) Schematic diagram of experiments for MPEP effects. The mice were treated with MPEP at 3 ​mg/kg/day by intraperitoneal injection for 4 weeks. (c) MPEP treatment reverses the effects of PFF injection on α-syn pathology. Representative pSer129 α-syn pathology images are shown in ipsilateral Cg, M, AI, LO, CPu, Thal, SN, and SC of PFF ​+ ​PBS or PFF ​+ ​MPEP groups. Scale bar, 50 ​μm. Percentage area occupied with α-syn pathology in ipsilateral regions. Heatmaps are representative of A53 α-syn Tg mice analyzed with PFF ​+ ​PBS (*n* ​= ​4), and PFF ​+ ​MPEP (*n* ​= ​4) groups at 31 days post-injection. ∗*P* ​< ​0.05 compared to PFF ​+ ​NCRS, unpaired *t*-test, two-tailed. (d) MPEP treatment inhibits neuronal activity as measured by c-Fos induction induced by PFF injection. *n* ​= ​4–5 per group. Representative c-Fos immunostaining images are shown in ipsilateral Cg, PrL, M, BLA, Au, Ect, and Ent of PFF ​+ ​PBS or PFF ​+ ​MPEP groups. Scale bar, 50 ​μm ∗*P* ​< ​0.05, compared to PFF ​+ ​NCRS, unpaired *t*-test, two-tailed. See also Supplementary Fig. 6 and Supplementary Fig. 7.

Neurons bearing α-syn aggregates are known to release α-syn, which then propagates to neighboring neurons [51]. Previous research has demonstrated that α-syn can bind to mGluR5 [52], and activation of mGluR5 results in c-Fos induction [53]. Based on these findings, we hypothesized that α-syn released from inclusion-bearing neurons activates mGluR5 in adjacent neurons to trigger c-Fos expression. RNAscope analysis confirmed substantial colocalization of c-Fos and mGluR5 mRNA in multiple brain regions (Fig. 5a and Supplementary Fig. 6). Pharmacological inhibition of mGluR5 with MPEP, a mGluR5 antagonist (Fig. 5b), reduced both pSer129 α-syn reactivity, particularly in the rostral regions (Fig. 5c, Supplementary Fig. 7a, and Supplementary Table 3), and c-Fos induction (Fig. 5d, Supplementary Fig. 7b, and Supplementary Table 4). These findings suggest that mGluR5 may mediate the induction of c-Fos by α-syn released from neurons bearing α-syn inclusions.

## Discussion

This study elucidates the complex reciprocal association between depression/anxiety-like behaviors and α-syn pathology in PD. Using combined animal models, we demonstrated that CRS-induced affective disturbances were exacerbated by α-syn propagation. While A53T α-syn overexpression alone did not modify susceptibility to CRS-induced depressive and anxiety-like behaviors in young mice, addition of PFF injection intensified these behavioral phenotypes, highlighting the necessity of α-syn propagation for symptom aggravation.

Previous studies have reported increased anxiety-like behaviors in A53T α-syn Tg mice at later stages of life, 14–15 months as noted by Kim et al. [54], and at 6 months, as reported by Li et al. [55] under basal conditions. The discrepancy with our findings, which used younger mice aged 2–3 months, suggests that the age of the mice significantly influences the manifestation of anxiety-like behaviors related to α-syn pathology in Tg models. This underscores the important role of age in modulating affective symptom expression associated with α-syn pathology.

CRS facilitated α-syn propagation, indicative of PD progression, and accelerated PFF-induced dopaminergic neurodegeneration. While PFF injection alone did not cause significant loss of TH-positive neurons in the substantia nigra within one month [47], CRS/PFF treatment resulted in reduced TH-positive cell counts in the substantia nigra and decreased striatal TH immunoreactivity. These findings parallel clinical reports linking depression to greater PD severity, reflected by higher Unified Parkinson’s Disease Rating Scale scores, advanced Hoehn and Yahr stages [7], and increased neuronal loss and gliosis in the substantia nigra compacta of depressed versus non-depressed patients [56].

In PD, α-syn spread from the olfactory bulb primarily involves the olfactory pathway and limbic system, contributing to hyposmia, anxiety, and memory deficits [57]. Anxiety in PD has been speculated to result from the deposition of α-syn in non-dopaminergic areas [8,58,59]. This study explored how neuronal activity links α-syn propagation and depression/anxiety-like behaviors. c-Fos expression served as a marker of neuronal activity. Acting through AP-1-dependent transcriptional programs, c-Fos regulates membrane, dendritic, and synaptic remodeling, forming feedback circuits that heighten excitability [60]. Persistent hyperactivity may in turn facilitate α-syn release and propagation, as reported in studies demonstrating activity-driven increases of extracellular and phosphorylated α-syn [21,22], as well as tau and Aβ spread in Alzheimer’s disease (AD) models [23,24]. CRS increased c-Fos expression in multiple regions, including the motor cortex, insular cortex, basolateral amygdaloid nucleus, and perirhinal cortex, in both PBS- and PFF-treated mice, with broader and stronger induction observed in the PFF group. Notably, even without CRS, PFF-treated mice exhibited elevated neuronal activity in the motor, sensory, and piriform cortices, as well as the basolateral amygdaloid, perirhinal, ectorhinal, and entorhinal cortices, compared with controls. The distribution of pSer129 α-syn-positive inclusions correlated with that of c-Fos-positive neurons, suggesting that α-syn inclusion formation and increased neural activity may act synergistically to potentiate each other. Unexpectedly, c-Fos-positive neurons showed limited colocalization with pSer129 α-syn-positive cell bodies, despite both being primarily excitatory neurons, indicating that the lack of overlap is not due to neuronal subtype differences. It has been reported that pSer129 α-syn-positive neurons are excitatory neurons [61], in which α-syn is highly expressed [62]. This pattern, consistent with observations in PD and DLB patient brains [63], supporting our data. These results suggest that LB-like inclusions bearing neurons may not be activated, but rather the neural activity of neighboring neurons near those with LB-like inclusions is increased. Likewise, hyperactive neurons were found exclusively near the plaques of Aβ deposition in an AD mouse model. The authors proposed that these hyperactive neurons are likely candidates to trigger this pathological activity [64], and Aβ pathology also promotes hyperexcitation and tonic hyperactivity in a large number of neurons in hippocampal and neocortical brain regions [65], suggesting that neurodegenerative diseases may share a common underlying mechanism, which aligns with our findings.

Administration of the c-Fos inhibitor T5224 effectively attenuated CRS-enhanced α-syn propagation and the associated exacerbation of depression/anxiety-like behaviors, supporting a bidirectional role of c-Fos induction in linking affective symptoms and α-syn spread. Notably, T5224 also reduced α-syn propagation in the absence of CRS. This aligns with reports that perampanel, an antiepileptic modulating neuronal activity, ameliorates α-syn pathology in PFF-inoculated WT mice [66], underscoring the influence of neural activity on the interplay between depression/anxiety behaviors and α-syn propagation. Although the focus was on neural activity inhibition via T5224, potential contributions of c-Fos downstream signaling pathways, including anti-inflammatory effects known from T5224 in others [67,68], cannot be excluded. Further research is necessary to delineate the specific roles of c-Fos downstream pathways in these processes.

The inter-neuronal spread of α-syn requires its extracellular release and subsequent uptake by neighboring neurons, primarily via receptor-mediated endocytosis, though tunneling nanotubes may also contribute [51]. Previous studies have demonstrated that mGluR5 gene transfer into the hippocampal CA1 region using a lentiviral vector resulted in increased α-syn accumulation and neurodegeneration in both single and α-syn/APP Tg mice [69]. Additionally, CTEP, a negative allosteric modulator of mGluR5, significantly reduced α-syn accumulation in SH-SY5Y cells [70]. Moreover, mGluR5 agonists have been shown to induce c-Fos expression [53]. Given prior evidence implicating mGluR5 in α-syn accumulation and neurodegeneration, we examined its role in α-syn propagation. mGluR5 inhibition with an antagonist suppressed c-Fos induction in neighboring neurons and reduced α-syn spread, particularly in rostral brain regions, consistent with its high expression in the striatum, hippocampus, and frontal cortex, and relatively lower expression in the cerebellum and pons/medulla [71,72]. These results suggest that mGluR5 contributes to c-Fos induction and facilitates α-syn propagation. As CRS has been reported to elevate hippocampal mGluR5 levels [73], CRS-induced changes in mGluR5 expression may further modulate α-syn spread, although this was not directly assessed here.

While our study advances understanding of the interplay between affective behaviors, neural activity, and α-syn propagation, limitations remain. Specifically, the exact neural networks mediating these interactions were not precisely identified, reflecting the complexity of region- and neuron-specific effects [74]. Furthermore, discrepancies with prior chronic stress models highlight variability in behavioral outcomes [75], and emphasize the need for more refined approaches to capture diverse physiological and molecular consequences. This variability is likely attributable to differences in stress paradigms, animal strains, and experimental conditions commonly observed in chronic stress-induced depression models [76]. In addition, only male mice were used throughout the study to minimize variability arising from hormonal fluctuations that influence stress responses and α-syn pathology. However, Clinical data indicate sex differences in the prevalence of depression and anxiety in PD, with women affected more frequently than men [77]. Accordingly, future work including both sexes will be required to determine whether sex-specific differences modulate the c-Fos-dependent mechanisms of α-syn propagation. Moreover, although modulation of c-Fos- or mGluR5-related signaling represents an intriguing therapeutic avenue, direct pharmacological inhibition of c-Fos may not be feasible because of its widespread expression and pleiotropic roles across multiple tissues. Instead, region-specific modulation of upstream pathways that regulate neuronal activity and c-Fos expression, such as mGluR5-mediated signaling, may offer safer and more precisely targeted strategies for intervention. Such approaches could enable more selective modulation of neural circuits implicated in both affective and α-syn pathology, ultimately improving translational relevance.

In conclusion, this study highlights a reciprocal association between depression/anxiety-like behaviors and α-syn pathology in PD, in which these factors appear to interact dynamically and their coexistence may contribute to disease progression. Modulation of c-Fos expression, notably via the inhibitor T5224, mitigated both behavioral deficits and α-syn spread, indicating its therapeutic potential for PD non-motor symptoms. mGluR5 further contributed to this interplay, underscoring the central role of neuronal activity. These findings advocate early, dual-target strategies against neuronal hyperactivity and α-syn pathology to slow PD progression and enhance quality of life.

## Submission declaration

The work described in this article has not been published previously or is under consideration for publication elsewhere. All authors have approved the submission of this article.

## Author contributions

**Soo-Jeong Kim:** Conceptualization, Methodology, Data curation, Investigation, Formal analysis, Validation, Visualization, Resources, Writing - original draft, Project administration, **Jae-Bong Kim:** Methodology, Formal analysis, Investigation, Validation, Visualization, Resources, **Seonghui Ham:** Methodology, Formal analysis, Investigation, Validation, Visualization, Resources, **Sang Myun Park:** Conceptualization, Methodology, Data curation, Investigation, Formal analysis, Funding acquisition, Resources, Writing - original draft, Writing - review & editing, Project administration, Supervision.

## Funding

This work was supported by the 10.13039/501100003725National Research Foundation of Korea (NRF) grants funded by the Korean government (Ministry of Science and ICT) (Grant No. RS-2019-NR040055, RS-2023-00262332, and RS-2025-00515932), and by a grant of the Korea Health Technology R&D Project through the Korea Health Industry Development Institute (KHIDI), funded by the Ministry of Health and Welfare, Republic of Korea (Grant No. RS-2024-00439928).

## Declaration of competing interest

The authors declare that they have no known competing financial interests or personal relationships that could have appeared to influence the work reported in this paper.
