# Supplementary material for: Uncovering the role of c-Fos in the bidirectional relationship between depression/anxiety behaviors and α-synuclein propagation in Parkinson’s disease
Source: Neurotherapeutics. 2025 Nov 26;23(1):e00807. doi: 10.1016/j.neurot.2025.e00807 (PMC12976557; doi:10.1016/j.neurot.2025.e00807)
Supplement: Multimedia component 1 [file mmc1.docx]

**Supplementary Materials**

**Uncovering the role of c-Fos in the bidirectional relationship between depression/anxiety behaviors and α-synuclein propagation in Parkinson's disease**

Soo-Jeong Kim^a^, Jae-Bong Kim^a^, Seonghui Ham^a,b,c^, and Sang Myun Park^a,b,c*^

^a^Center for Convergence Research of Neurological Disorders, Ajou University School of Medicine, Suwon, Korea, ^b^Department of Pharmacology, Ajou University School of Medicine, Suwon, Korea, ^c^Neuroscience Graduate Program, Department of Biomedical Sciences, Ajou University School of Medicine, Suwon, Korea,

* Corresponding authors at: Dr. Sang Myun Park, Department of Pharmacology, Ajou University School of Medicine, 164, Worldcup-ro, Yeongtong-gu, Suwon 16499, Korea.

E-mail address: [sangmyun@ajou.ac.kr](mailto:sangmyun@ajou.ac.kr) (S.M. Park). Tel: 82-31-219-5063, Fax: 82-31-219-5069.

Running title: c-Fos mediates depression/anxiety and α-synuclein propagation in Parkinson's disease

**Supplementary Figures**

**
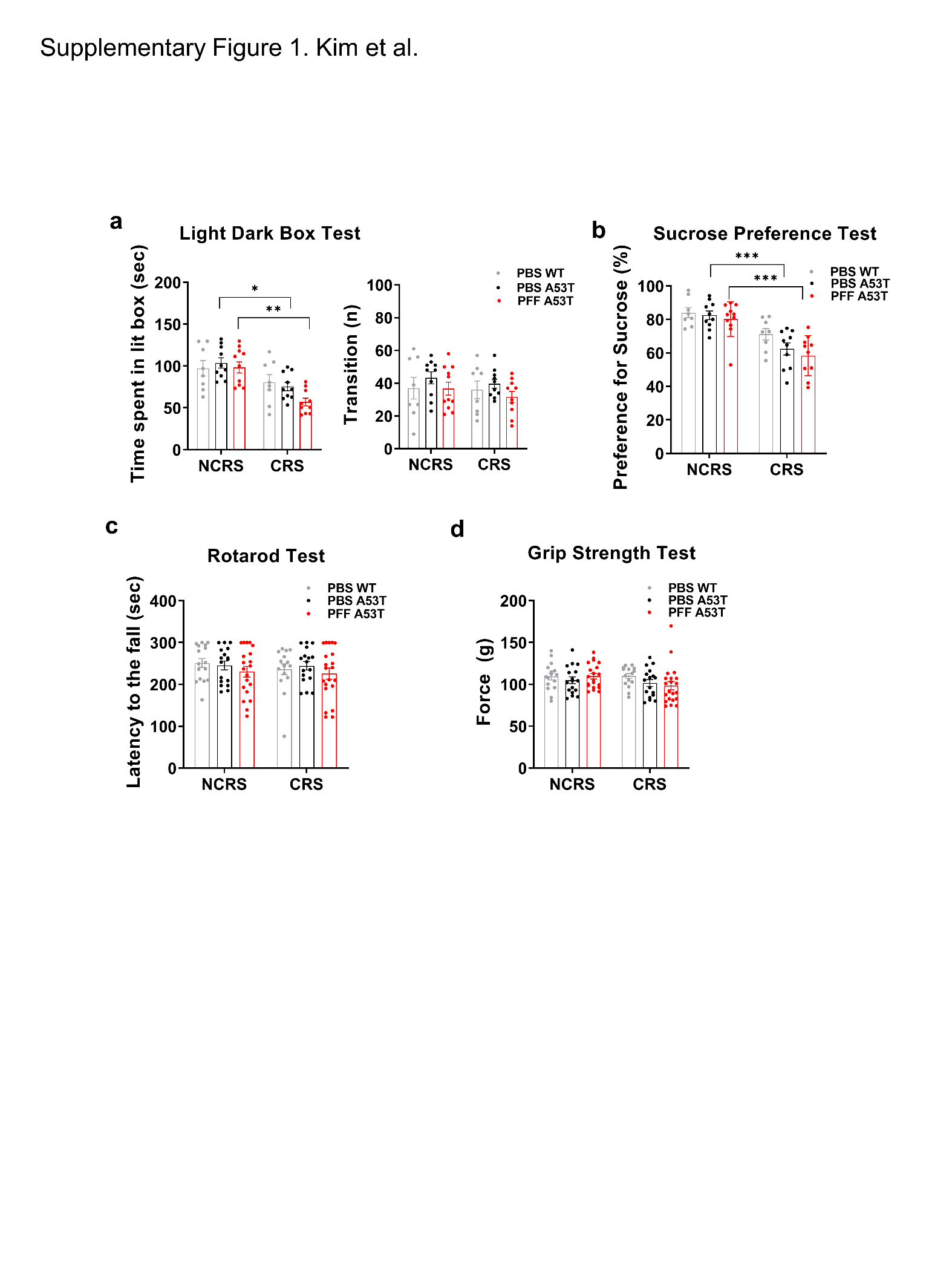
**

**Supplementary Fig. 1** **Behavioral results of PFF-injected mice under CRS treatment** **in the light-dark box, sucrose preference, rotarod, and grip strength tests.**

**(a)** Quantification of the total time spent in the light compartment and transition of the light-dark box. **(b)** Anhedonic behavior in the sucrose preference test is shown. **(c)** In the rotarod test, the latency to fall off the rod, which evaluates motor coordination and balance, is presented. **(d)** In the forelimb grip strength test, the force in grams used to measure neuromuscular strength is recorded. n=8–11 per group for A and B, n=16–21 per group for C and D. *** p < 0.001, ** p < 0.01, * p < 0.05, two-way ANOVA with, Tukey's multiple comparison tests.


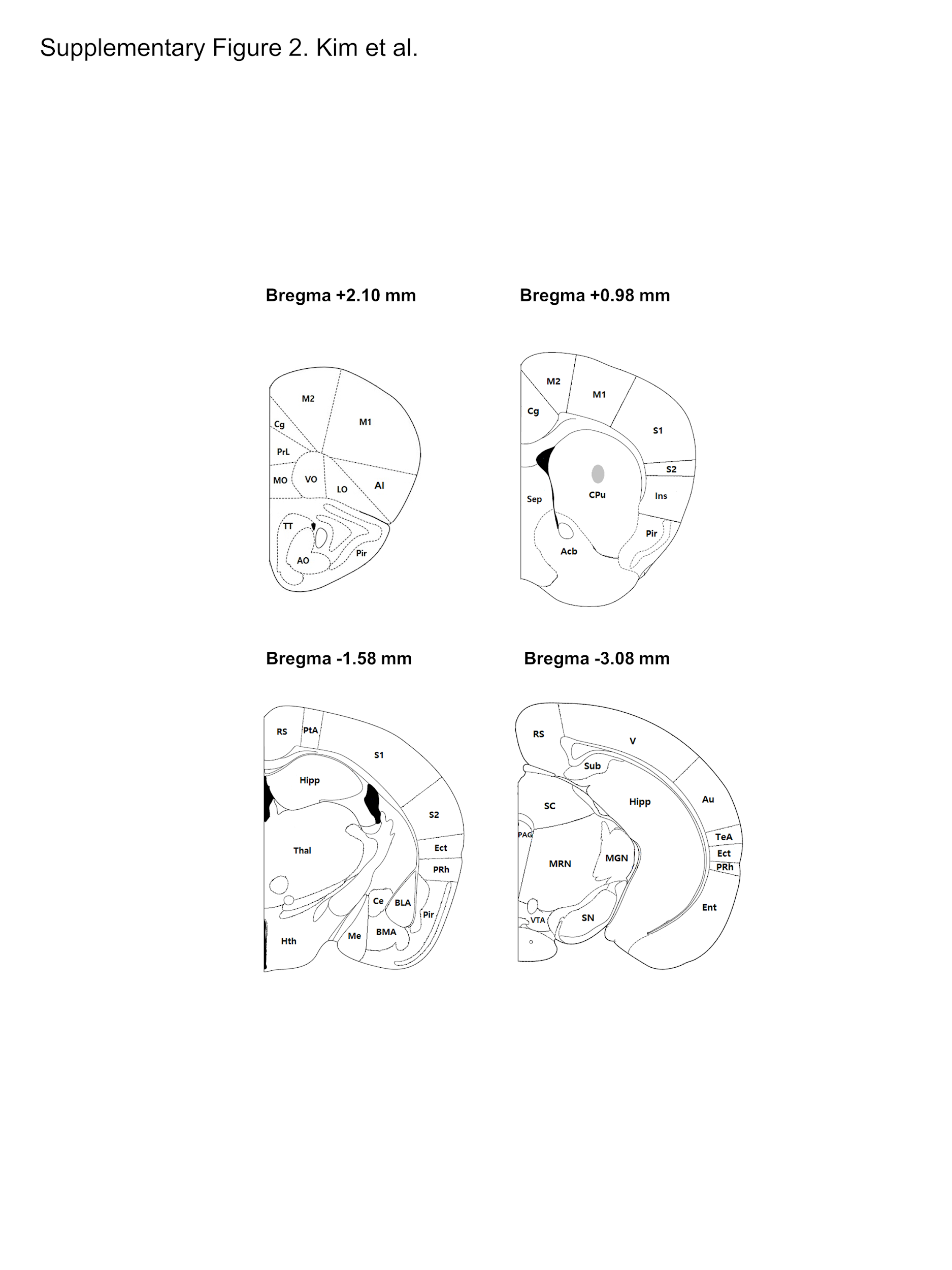


**Supplementary Fig. 2 Schematic images of the regions annotated for pathology measures and used in subsequent analysis.**

Bolded regions were analyzed. Abbreviations : Acb = nucleus accumbens; AI = agranular insular cortex; AO = anterior olfactory nucleus; Au = auditory cortex; BLA = basolateral amygdaloid nucleus; BMA = basomedial amygdaloid nucleus; Ce = central amygdaloid nucleus; Cg = cingulate cortex; Cpu = caudate putamen (striatum); Ect = ectorhinal cortex; Ent = entorhinal cortex; Hipp = hippocampus; Hth = hypothalamus; Ins = insular cortex; LO = lateral orbital cortex; Me = medial amygdaloid nucleus; MGN = medial geniculate nucleus; M1 = motor cortex 1; M2 = motor cortex 2; MO = medial orbital cortex; MRN = midbrain red nucleus; PAG = periaqueductal gray;  Pir = piriform cortex;  PrL = prelimbic cortex; PRh = perirhinal cortex; PtA = parietal association cortex; RS = retrosplenial cortex; S1 = somatosensory cortex 1; S2 = somatosensory cortex 2; SC = superior colliculus; Sep = septal; SN = substantia nigra; Sub = subiculum; TeA = temporal association cortex; Thal = thalamic nuclei; TT = tenia tecta; V = visual cortex; VO = ventral orbital cortex; VTA = ventral tegmental area


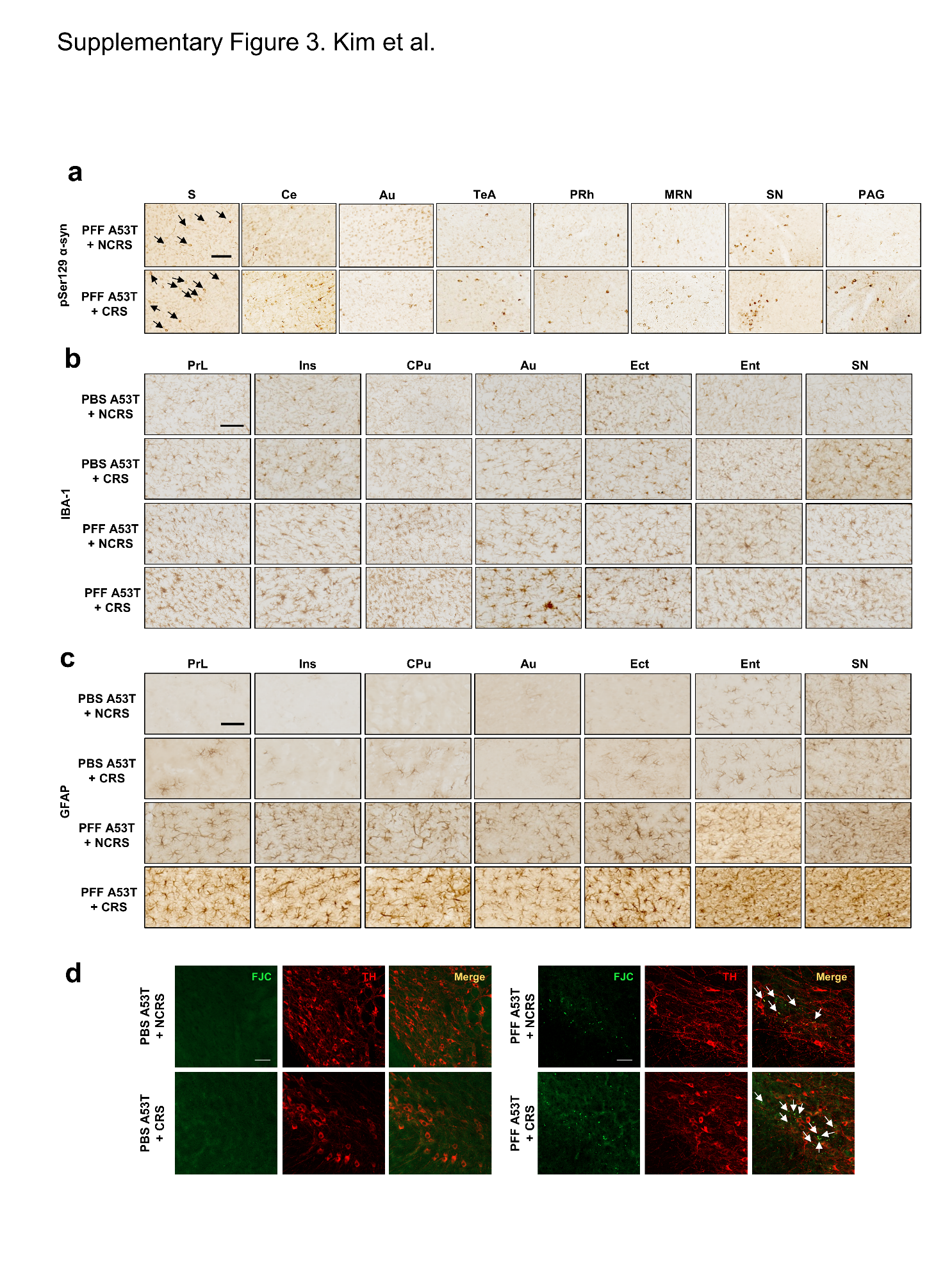


**Supplementary Fig. 3** **CRS triggers α-syn propagation, microgliosis, and astrogliosis induced by PFF injection.**

**(a)** Pathology (pSer129) detected in numerous brain regions (S, Ce, Au, TeA, PRh, MRN, SN, and PAG regions) of PFF + NCRS, and PFF + CRS groups in ipsilateral structures. Abbreviations are available in Fig. S2. Scale bar, 50μm. **(b)** Representative immunostaining for Iba1 in the ipsilateral PrL, Ins, CPu, Au, Ect, Ent, and SN regions of PBS + NCRS, PBS + CRS, PFF + NCRS, and PFF + CRS groups. Scale bar, 50μm. **(c)** Representative immunostaining for GFAP in the ipsilateral PrL, Ins, CPu, Au, Ect, Ent, and SN regions of PBS + NCRS, PBS + CRS, PFF + NCRS, and PFF + CRS groups. Scale bar, 50μm.  **(d)** Double immunofluorescence staining of TH (red) and FJC (green) in the SN of PBS + NCRS, PBS + CRS, PFF + NCRS, and PFF + CRS groups; merged images at 4weeks of CRS slightly induced neurodegeneration in the SN of A53Tg mice injected with PFF.


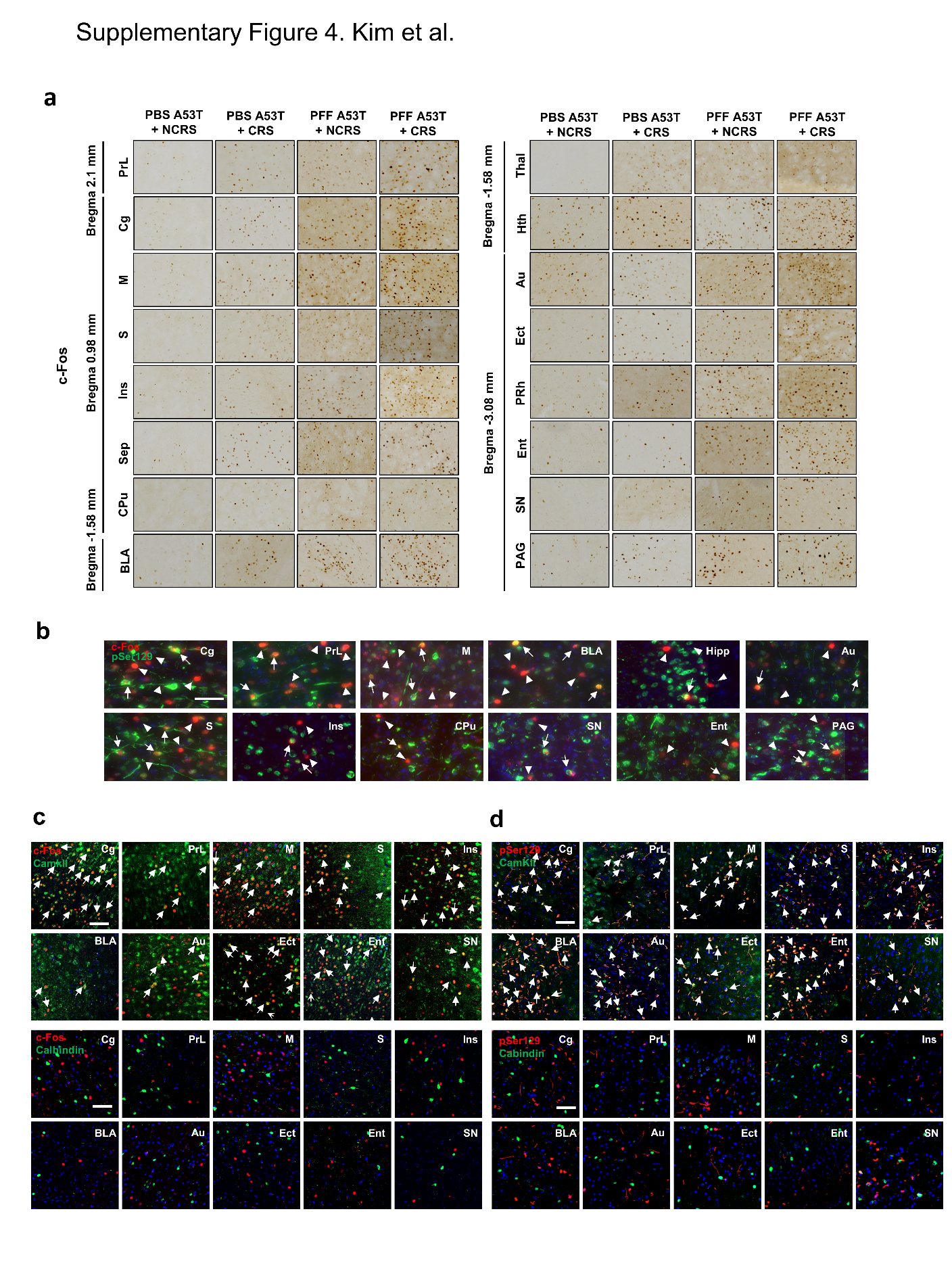


**Supplementary Fig. 4 The hyperactive neurons are likely candidates to trigger α-syn pathology.**

**(a)** Immunohistochemistry of c-Fos in the ipsilateral PrL, Cg, M, S, Ins, Sep, CPu, BLA, Thal, Hth, Au, Ect, PRh, Ent, SN, and PAG regions of PBS + NCRS, PBS + CRS, PFF + NCRS, and PFF + CRS groups. **(b**) Double-labeling immunofluorescence disclosed co-localization of c-Fos and pser129-positive inclusions in the majority but not all neurons containing Lowy bodies in the brains of PFF-injected A53T mice. **(c)** Representative confocal images showing c-Fos (red), Ca2+/calmodulin-dependent protein kinase II excitatory neurons (CaMKII) (green), and calbindin (CB) inhibitory neurons (green) in the M. Scale bars, 50 μm. c-Fos primarily localized CaMKII and appeared to be excluded from CB neurons. **(d)** pSer129-α-syn-positive inclusions were localized almost exclusively to excitatory CaMKII neurons, and the Inhibitory neurons expressing, CB showed minimal to no pSer129-α-syn-positive inclusions.


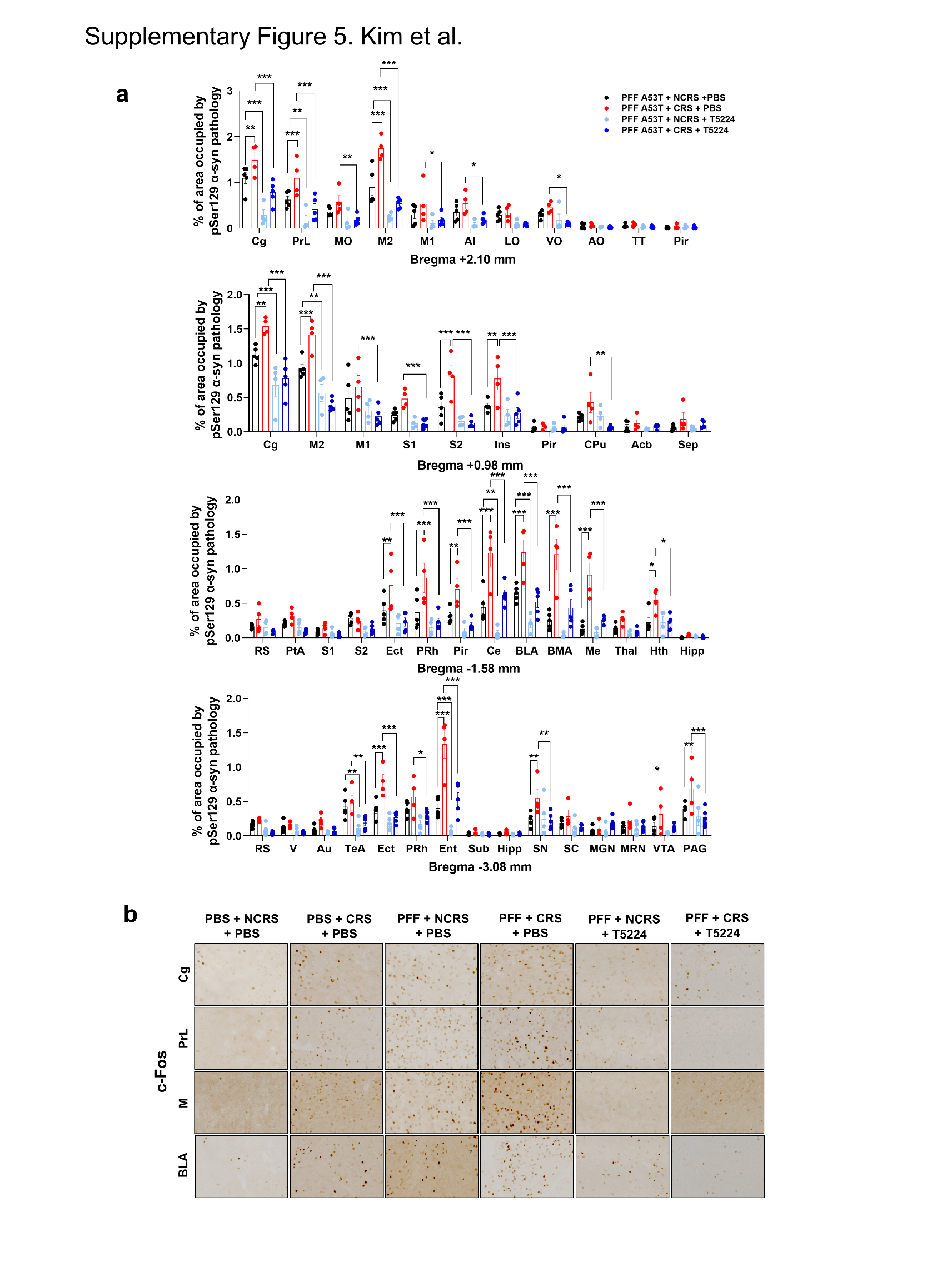


**Supplementary Fig. 5 T5224 alleviates α-syn propagation associated with c-Fos induction in PFF-injected A53 Tg mice.**

**(a)** Quantitative analysis of pSer129 α-syn-immunoreactive inclusions in numerous brain regions of PFF + NCRS + PBS (n=5), PFF + CRS + PBS (n=4), PFF + NCRS +T5224 (n=4) and PFF + CRS + T5224 (n=5) A53Tg mice in ipsilateral structures. *** p < 0.001, ** p < 0.01, * p < 0.05, two-way ANOVA with, Tukey's multiple comparison test**. (b)** Representative c-Fos images are shown in Cg, PrL, M, and BLA of PBS + NCRS + PBS, PBS + CRS + PBS, PFF + NCRS + PBS, PFF + CRS + PBS, PFF + NCRS +T5224 and PFF + CRS + T5224 A53Tg mice. Scale bars, 50 μm.


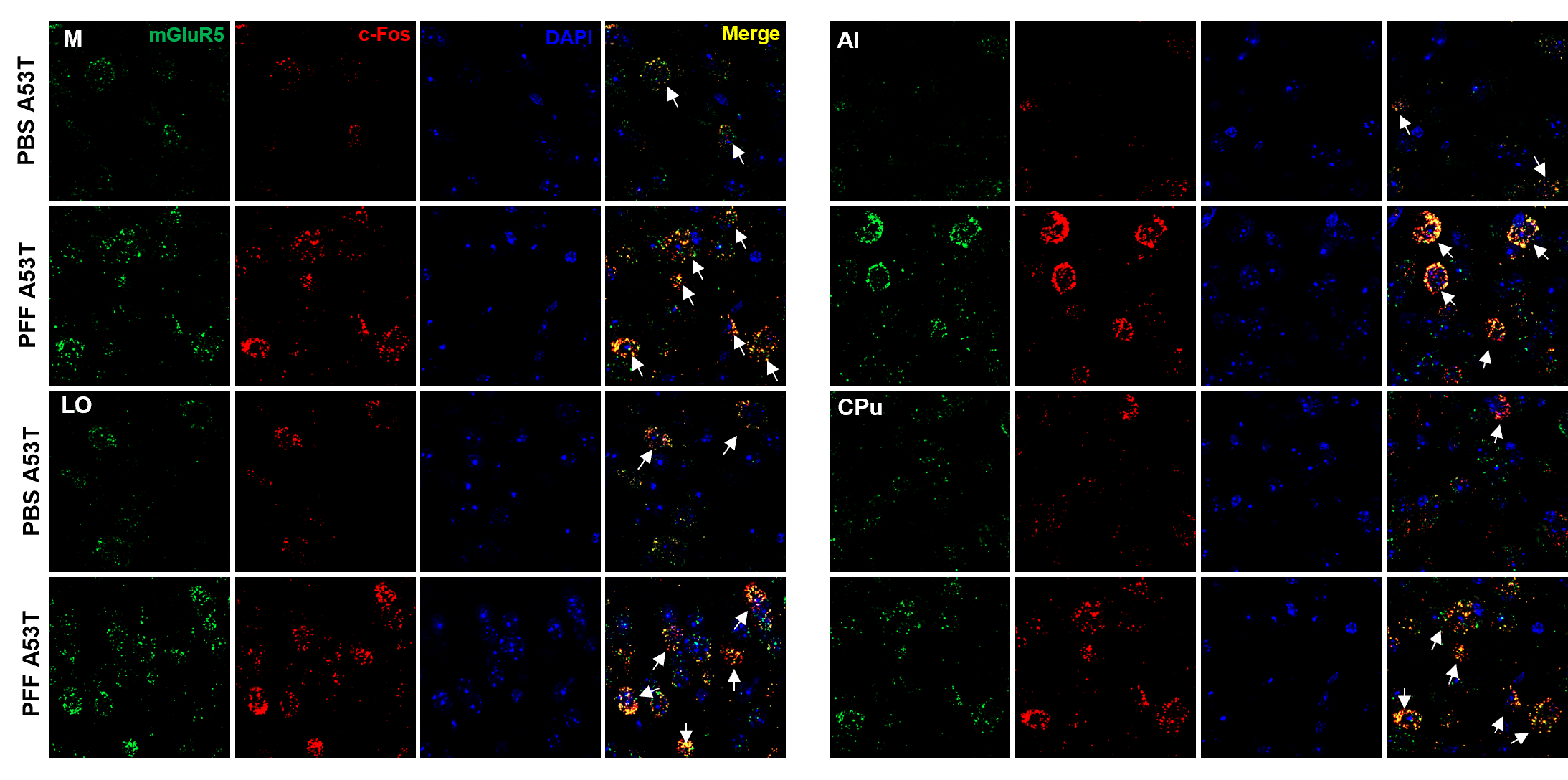


**Supplementary Fig. 6 mGluR5 mRNA is predominantly expressed in c-Fos-positive cells.**

Expression and colocalization of mGluR5 mRNA in M, AI, LO, and CPu. Scale bar, 20 μm.

**
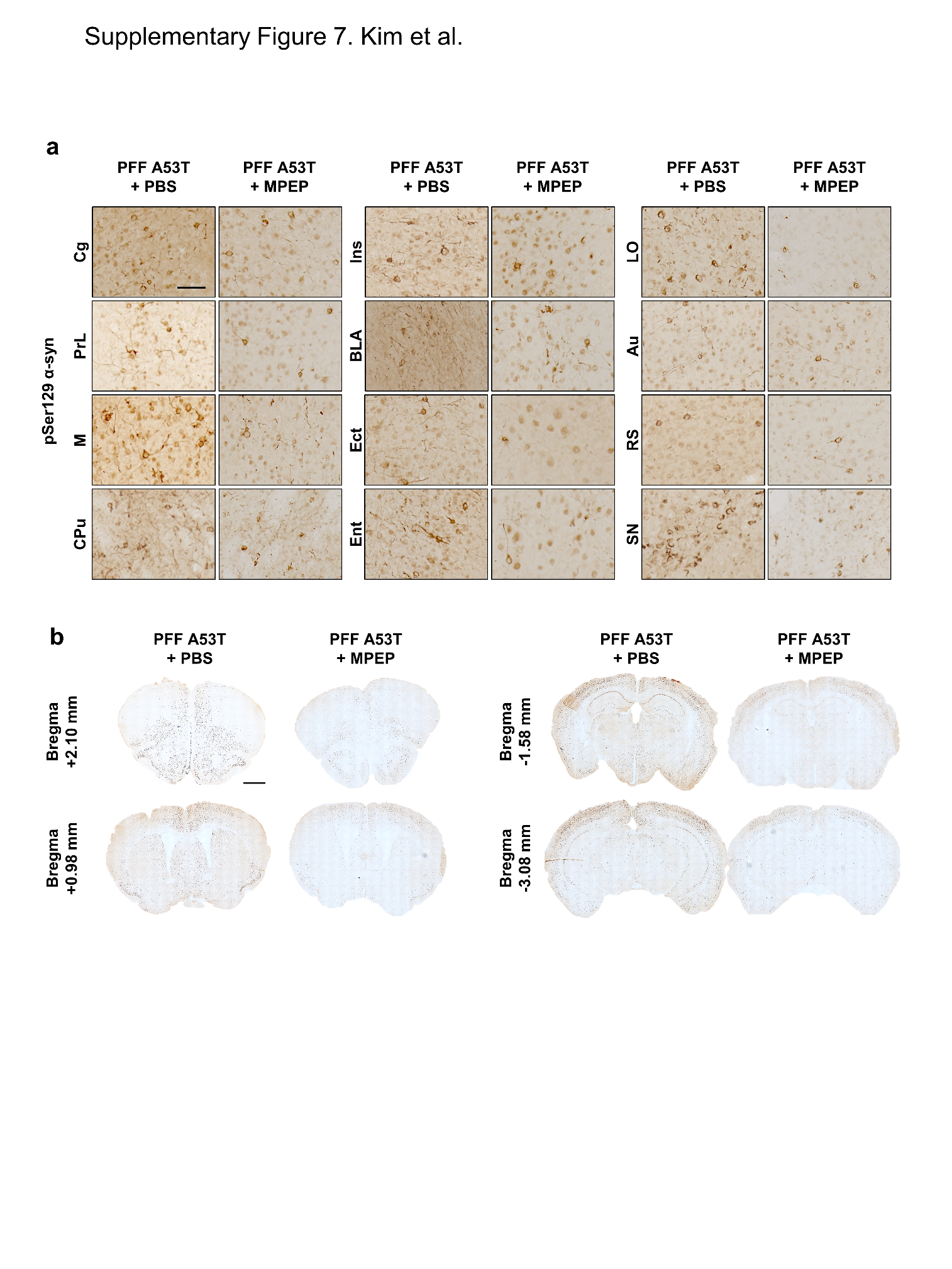
**

**Supplementary Fig. 7 MPEP alleviates α-syn propagation associated with c-Fos induction in PFF-injected A53 Tg mice*.***

**(a)** Representative pSer129 α-syn images are shown in Cg, PrL, M, CPu, Ins, BLA, Ect, Ent, LO, Au, RS, and SN of PFF + PBS and PFF + MPEP A53Tg mice. **(b)** Representative coronal brain sections from PFF + PBS and PFF + MPEP A53Tg mice at four positions of the brain stained for c-Fos under PBS (left) and MPEP (right) treatment. Scale bars represent 1 mm.

**Supplementary Table**

| Bregma | Regions | PFF A53T + NCRS | | | | | | | | | | | | | | PFF A53T + CRS | | | | | | | | | | | | |
| --- | --- | --- | --- | --- | --- | --- | --- | --- | --- | --- | --- | --- | --- | --- | --- | --- | --- | --- | --- | --- | --- | --- | --- | --- | --- | --- | --- | --- |
|  |  | 1 | 2 | 3 | 4 | 5 | 6 | 7 | 8 | 9 | 10 | 11 | 12 | Mean | SEM | 1 | 2 | 3 | 4 | 5 | 6 | 7 | 8 | 9 | 10 | 11 | Mean | SEM |
| 2.1 | Cg | 0.25 | 0.23 | 0.07 | 0.17 | 0.12 | 0.20 | 0.24 | 0.27 | 0.26 | 1.10 | 0.63 | 1.17 | 0.39 | 0.11 | 0.4 | 0.42 | 0.3 | 0.36 | 0.59 | 0.51 | 0.36 | 1.34 | 1.1 | 1.78 | 1.76 | 0.81 | 0.17 |
| 2.1 | PrL | 0.09 | 0.05 | 0.03 | 0.03 | 0.07 | 0.18 | 0.16 | 0.23 | 0.18 | 0.81 | 0.43 | 0.51 | 0.23 | 0.07 | 0.22 | 0.36 | 0.43 | 0.35 | 0.18 | 0.24 | 0.2 | 0.83 | 0.72 | 1.26 | 1.58 | 0.58 | 0.14 |
| 2.1 | MO | 0.06 | 0.08 | 0.01 | 0.04 | 0.06 | 0.13 | 0.16 | 0.10 | 0.12 | 0.44 | 0.30 | 0.29 | 0.15 | 0.04 | 0.08 | 0.34 | 0.22 | 0.41 | 0.28 | 0.1 | 0.18 | 0.35 | 0.42 | 0.54 | 0.98 | 0.35 | 0.08 |
| 2.1 | M2 | 0.33 | 0.55 | 0.19 | 0.43 | 0.64 | 0.26 | 0.28 | 0.42 | 0.33 | 0.63 | 0.64 | 0.59 | 0.44 | 0.05 | 0.58 | 0.7 | 0.63 | 0.79 | 0.62 | 0.58 | 0.57 | 1.59 | 1.79 | 1.51 | 2.07 | 1.04 | 0.17 |
| 2.1 | M1 | 0.12 | 0.20 | 0.22 | 0.17 | 0.28 | 0.35 | 0.26 | 0.16 | 0.21 | 0.10 | 0.38 | 0.05 | 0.21 | 0.03 | 0.92 | 0.81 | 0.69 | 0.88 | 0.45 | 0.15 | 0.38 | 0.49 | 1.15 | 0.18 | 0.31 | 0.58 | 0.10 |
| 2.1 | AI | 0.08 | 0.22 | 0.23 | 0.11 | 0.16 | 0.41 | 0.37 | 0.19 | 0.15 | 0.14 | 0.37 | 0.49 | 0.24 | 0.04 | 0.52 | 0.23 | 0.63 | 0.68 | 0.29 | 0.1 | 0.21 | 0.36 | 0.84 | 0.32 | 0.63 | 0.44 | 0.07 |
| 2.1 | LO | 0.08 | 0.15 | 0.11 | 0.05 | 0.06 | 0.22 | 0.29 | 0.12 | 0.20 | 0.15 | 0.36 | 0.29 | 0.17 | 0.03 | 0.22 | 0.36 | 0.27 | 0.49 | 0.23 | 0.11 | 0.15 | 0.26 | 0.5 | 0.16 | 0.44 | 0.29 | 0.04 |
| 2.1 | VO | 0.07 | 0.19 | 0.09 | 0.09 | 0.06 | 0.10 | 0.20 | 0.10 | 0.33 | 0.28 | 0.17 | 0.33 | 0.17 | 0.03 | 0.15 | 0.34 | 0.23 | 0.36 | 0.39 | 0.15 | 0.18 | 0.37 | 0.51 | 0.34 | 0.59 | 0.33 | 0.04 |
| 2.1 | AO | 0.00 | 0.03 | 0.02 | 0.04 | 0.11 | 0.04 | 0.15 | 0.01 | 0.07 | 0.06 | 0.00 | 0.10 | 0.05 | 0.01 | 0.1 | 0.29 | 0.22 | 0.07 | 0.03 | 0.04 | 0.03 | 0.07 | 0.04 | 0.03 | 0.12 | 0.09 | 0.03 |
| 2.1 | TT | 0.02 | 0.09 | 0.05 | 0.05 | 0.25 | 0.13 | 0.17 | 0.02 | 0.08 | 0.04 | 0.04 | 0.03 | 0.08 | 0.02 | 0.1 | 0.28 | 0.07 | 0.16 | 0.16 | 0.02 | 0.11 | 0.08 | 0.03 | 0.1 | 0.13 | 0.11 | 0.02 |
| 2.1 | Pir | 0.02 | 0.03 | 0.01 | 0.02 | 0.41 | 0.06 | 0.09 | 0.01 | 0.08 | 0.03 | 0.02 | 0.00 | 0.07 | 0.03 | 0.13 | 0.49 | 0.17 | 0.1 | 0.04 | 0.05 | 0.08 | 0.01 | 0.02 | 0.01 | 0.11 | 0.11 | 0.04 |
| 0.98 | Cg | 0.40 | 0.29 | 0.08 | 0.15 | 0.24 | 0.29 | 0.29 | 0.30 | 0.24 | 1.11 | 0.20 | 0.97 | 0.38 | 0.09 | 0.24 | 0.2 | 0.49 | 0.36 | 0.48 | 0.44 | 0.5 | 1.61 | 1.67 | 1.46 | 1.43 | 0.81 | 0.18 |
| 0.98 | M2 | 0.46 | 0.55 | 0.35 | 0.33 | 0.54 | 0.34 | 0.20 | 0.21 | 0.23 | 0.80 | 0.60 | 0.92 | 0.46 | 0.07 | 0.47 | 0.29 | 0.46 | 0.51 | 0.77 | 0.62 | 0.47 | 1.62 | 1.51 | 1.11 | 1.44 | 0.84 | 0.15 |
| 0.98 | M1 | 0.39 | 0.31 | 0.36 | 0.20 | 0.32 | 0.33 | 0.20 | 0.18 | 0.17 | 0.33 | 0.68 | 0.28 | 0.31 | 0.04 | 0.53 | 0.32 | 0.22 | 0.76 | 0.35 | 0.44 | 0.49 | 0.73 | 1.08 | 0.32 | 0.51 | 0.52 | 0.08 |
| 0.98 | S1 | 0.22 | 0.26 | 0.22 | 0.07 | 0.11 | 0.32 | 0.05 | 0.04 | 0.08 | 0.28 | 0.14 | 0.18 | 0.16 | 0.03 | 0.27 | 0.24 | 0.1 | 0.46 | 0.12 | 0.17 | 0.3 | 0.4 | 0.55 | 0.35 | 0.63 | 0.33 | 0.05 |
| 0.98 | S2 | 0.08 | 0.17 | 0.56 | 0.05 | 0.09 | 0.32 | 0.02 | 0.07 | 0.10 | 0.24 | 0.12 | 0.35 | 0.18 | 0.05 | 0.62 | 0.1 | 0.11 | 0.34 | 0.11 | 0.11 | 0.25 | 0.86 | 0.81 | 0.44 | 1.18 | 0.45 | 0.11 |
| 0.98 | Ins | 0.09 | 0.17 | 0.33 | 0.11 | 0.11 | 0.40 | 0.12 | 0.07 | 0.18 | 0.28 | 0.37 | 0.36 | 0.22 | 0.04 | 0.74 | 0.87 | 0.12 | 0.58 | 0.3 | 0.11 | 0.41 | 0.96 | 0.39 | 0.35 | 0.7 | 0.50 | 0.09 |
| 0.98 | Pir | 0.02 | 0.03 | 0.10 | 0.25 | 0.61 | 0.05 | 0.08 | 0.01 | 0.05 | 0.02 | 0.05 | 0.01 | 0.11 | 0.05 | 0.12 | 0.91 | 0.02 | 0.17 | 0.16 | 0.03 | 0.39 | 0.09 | 0.07 | 0.02 | 0.12 | 0.19 | 0.08 |
| 0.98 | Cpu | 0.06 | 0.09 | 0.11 | 0.08 | 0.15 | 0.13 | 0.04 | 0.08 | 0.10 | 0.15 | 0.17 | 0.21 | 0.11 | 0.01 | 0.12 | 0.41 | 0.10 | 0.17 | 0.16 | 0.23 | 0.39 | 0.09 | 0.07 | 0.45 | 0.12 | 0.21 | 0.04 |
| 0.98 | Acb | 0.03 | 0.02 | 0.02 | 0.05 | 0.09 | 0.04 | 0.01 | 0.01 | 0.03 | 0.15 | 0.01 | 0.01 | 0.04 | 0.01 | 0.02 | 0.46 | 0.03 | 0.04 | 0.02 | 0.03 | 0.05 | 0.1 | 0.03 | 0.07 | 0.28 | 0.10 | 0.04 |
| 0.98 | Sep | 0.07 | 0.04 | 0.01 | 0.06 | 0.09 | 0.11 | 0.04 | 0.06 | 0.12 | 0.06 | 0.04 | 0.05 | 0.06 | 0.01 | 0.02 | 0.61 | 0.25 | 0.04 | 0.04 | 0.08 | 0.06 | 0.12 | 0.05 | 0.11 | 0.47 | 0.17 | 0.06 |
| -1.58 | RS | 0.22 | 0.22 | 0.11 | 0.14 | 0.11 | 0.13 | 0.02 | 0.06 | 0.03 | 0.12 | 0.19 | 0.16 | 0.13 | 0.02 | 0.24 | 0.15 | 0.14 | 0.25 | 0.14 | 0.15 | 0.2 | 0.34 | 0.14 | 0.1 | 0.5 | 0.21 | 0.04 |
| -1.58 | PtA | 0.14 | 0.20 | 0.12 | 0.12 | 0.12 | 0.28 | 0.01 | 0.14 | 0.08 | 0.16 | 0.22 | 0.21 | 0.15 | 0.02 | 0.27 | 0.12 | 0.14 | 0.31 | 0.1 | 0.18 | 0.15 | 0.29 | 0.35 | 0.19 | 0.44 | 0.23 | 0.03 |
| -1.58 | S1 | 0.04 | 0.15 | 0.06 | 0.10 | 0.07 | 0.08 | 0.01 | 0.03 | 0.02 | 0.04 | 0.06 | 0.06 | 0.06 | 0.01 | 0.11 | 0.08 | 0.09 | 0.12 | 0.03 | 0.06 | 0.08 | 0.18 | 0.22 | 0.04 | 0.14 | 0.10 | 0.02 |
| -1.58 | S2 | 0.12 | 0.40 | 0.15 | 0.27 | 0.26 | 0.08 | 0.04 | 0.08 | 0.04 | 0.18 | 0.32 | 0.22 | 0.18 | 0.03 | 0.23 | 0.16 | 0.22 | 0.16 | 0.06 | 0.15 | 0.14 | 0.23 | 0.38 | 0.11 | 0.27 | 0.19 | 0.03 |
| -1.58 | Ect | 0.16 | 0.29 | 0.16 | 0.31 | 0.12 | 0.11 | 0.08 | 0.15 | 0.05 | 0.27 | 0.34 | 0.20 | 0.19 | 0.03 | 0.25 | 0.14 | 0.49 | 0.18 | 0.14 | 0.23 | 0.13 | 0.97 | 1.22 | 0.46 | 0.42 | 0.42 | 0.11 |
| -1.58 | PRh | 0.19 | 0.51 | 0.20 | 0.33 | 0.14 | 0.07 | 0.20 | 0.07 | 0.05 | 0.19 | 0.26 | 0.15 | 0.20 | 0.04 | 0.26 | 0.22 | 0.44 | 0.19 | 0.22 | 0.24 | 0.25 | 0.97 | 1.42 | 0.57 | 0.51 | 0.48 | 0.12 |
| -1.58 | Pir | 0.04 | 0.16 | 0.09 | 0.23 | 0.56 | 0.14 | 0.07 | 0.08 | 0.09 | 0.31 | 0.32 | 0.22 | 0.19 | 0.04 | 0.14 | 0.22 | 0.1 | 0.24 | 0.1 | 0.15 | 0.48 | 0.75 | 0.52 | 0.46 | 1.1 | 0.39 | 0.10 |
| -1.58 | Ce | 0.02 | 0.07 | 0.03 | 0.07 | 0.04 | 0.10 | 0.01 | 0.13 | 0.11 | 0.50 | 0.28 | 0.32 | 0.14 | 0.04 | 0.04 | 0.13 | 0.07 | 0.44 | 0.23 | 0.59 | 0.14 | 1.29 | 0.63 | 1.46 | 1.53 | 0.60 | 0.17 |
| -1.58 | BLA | 0.08 | 0.15 | 0.07 | 0.16 | 0.10 | 0.18 | 0.04 | 0.19 | 0.14 | 0.65 | 0.49 | 0.71 | 0.25 | 0.07 | 0.23 | 0.28 | 0.44 | 0.23 | 0.25 | 0.45 | 0.25 | 1.55 | 0.8 | 1.53 | 1.07 | 0.64 | 0.16 |
| -1.58 | BMA | 0.05 | 0.03 | 0.14 | 0.24 | 0.15 | 0.11 | 0.07 | 0.06 | 0.12 | 0.17 | 0.11 | 0.33 | 0.13 | 0.02 | 0.04 | 0.22 | 0.03 | 0.17 | 0.1 | 0.26 | 0.14 | 1.33 | 0.58 | 1.62 | 1.31 | 0.53 | 0.18 |
| -1.58 | Me | 0.08 | 0.03 | 0.03 | 0.04 | 0.15 | 0.11 | 0.10 | 0.06 | 0.09 | 0.17 | 0.09 | 0.06 | 0.08 | 0.01 | 0.08 | 0.12 | 0.04 | 0.07 | 0.05 | 0.24 | 0.13 | 1.22 | 0.57 | 0.7 | 1.17 | 0.40 | 0.14 |
| -1.58 | Thal | 0.04 | 0.07 | 0.02 | 0.03 | 0.02 | 0.10 | 0.01 | 0.05 | 0.00 | 0.07 | 0.10 | 0.11 | 0.05 | 0.01 | 0.16 | 0.06 | 0.09 | 0.1 | 0.1 | 0.08 | 0.11 | 0.29 | 0.26 | 0.16 | 0.38 | 0.16 | 0.03 |
| -1.58 | Hth | 0.03 | 0.10 | 0.01 | 0.02 | 0.01 | 0.20 | 0.01 | 0.14 | 0.06 | 0.16 | 0.15 | 0.13 | 0.09 | 0.02 | 0.06 | 0.1 | 0.12 | 0.12 | 0.09 | 0.13 | 0.07 | 0.51 | 0.32 | 0.66 | 0.69 | 0.26 | 0.07 |
| -1.58 | Hipp | 0.04 | 0.13 | 0.00 | 0.01 | 0.00 | 0.03 | 0.01 | 0.02 | 0.02 | 0.00 | 0.01 | 0.00 | 0.02 | 0.01 | 0.02 | 0.04 | 0.07 | 0.01 | 0.02 | 0.01 | 0.01 | 0.03 | 0.06 | 0.04 | 0.03 | 0.03 | 0.01 |
| -3.08 | RS | 0.08 | 0.00 | 0.02 | 0.04 | 0.01 | 0.03 | 0.08 | 0.03 | 0.00 | 0.10 | 0.19 | 0.15 | 0.06 | 0.02 | 0.06 | 0.02 | 0.05 | 0.18 | 0.19 | 0.13 | 0.08 | 0.2 | 0.2 | 0.24 | 0.26 | 0.15 | 0.02 |
| -3.08 | V | 0.03 | 0.06 | 0.01 | 0.02 | 0.02 | 0.13 | 0.08 | 0.01 | 0.01 | 0.08 | 0.07 | 0.32 | 0.07 | 0.03 | 0.05 | 0.04 | 0.11 | 0.14 | 0.02 | 0.07 | 0.06 | 0.18 | 0.34 | 0.23 | 0.11 | 0.12 | 0.03 |
| -3.08 | Au | 0.20 | 0.06 | 0.02 | 0.07 | 0.02 | 0.16 | 0.11 | 0.09 | 0.04 | 0.08 | 0.06 | 0.13 | 0.09 | 0.02 | 0.33 | 0.26 | 0.24 | 0.52 | 0.35 | 0.64 | 0.14 | 0.21 | 0.11 | 0.21 | 0.15 | 0.29 | 0.05 |
| -3.08 | TeA | 0.10 | 0.02 | 0.06 | 0.05 | 0.07 | 0.25 | 0.09 | 0.07 | 0.00 | 0.37 | 0.23 | 0.51 | 0.15 | 0.05 | 0.17 | 0.1 | 0.29 | 0.47 | 0.1 | 0.24 | 0.19 | 0.78 | 0.32 | 0.51 | 0.32 | 0.32 | 0.06 |
| -3.08 | Ect | 0.11 | 0.01 | 0.04 | 0.06 | 0.20 | 0.37 | 0.05 | 0.09 | 0.06 | 0.41 | 0.21 | 0.37 | 0.17 | 0.04 | 0.08 | 0.17 | 0.22 | 0.86 | 0.07 | 0.3 | 0.38 | 1.28 | 0.59 | 0.8 | 0.48 | 0.48 | 0.11 |
| -3.08 | PRh | 0.06 | 0.02 | 0.04 | 0.02 | 0.24 | 0.17 | 0.04 | 0.02 | 0.07 | 0.25 | 0.48 | 0.35 | 0.15 | 0.04 | 0.18 | 0.18 | 0.27 | 0.7 | 0.14 | 0.21 | 0.28 | 0.87 | 0.25 | 0.45 | 0.69 | 0.38 | 0.08 |
| -3.08 | Ent | 0.03 | 0.04 | 0.02 | 0.00 | 0.21 | 0.19 | 0.07 | 0.08 | 0.02 | 0.27 | 0.14 | 0.57 | 0.14 | 0.05 | 0.09 | 0.21 | 0.24 | 0.45 | 0.02 | 0.35 | 0.34 | 1.58 | 0.74 | 1.41 | 1.61 | 0.64 | 0.18 |
| -3.08 | Sub | 0.02 | 0.01 | 0.00 | 0.00 | 0.01 | 0.04 | 0.00 | 0.00 | 0.00 | 0.05 | 0.03 | 0.05 | 0.02 | 0.01 | 0.03 | 0.02 | 0.05 | 0.05 | 0.06 | 0.04 | 0.01 | 0.02 | 0.03 | 0.03 | 0.01 | 0.03 | 0.01 |
| -3.08 | Hipp | 0.02 | 0.02 | 0.02 | 0.10 | 0.03 | 0.06 | 0.00 | 0.02 | 0.01 | 0.01 | 0.01 | 0.03 | 0.03 | 0.01 | 0.03 | 0.11 | 0.12 | 0.05 | 0.02 | 0.014 | 0.03 | 0.02 | 0.02 | 0.09 | 0.07 | 0.05 | 0.01 |
| -3.08 | SN | 0.09 | 0.03 | 0.02 | 0.07 | 0.06 | 0.04 | 0.04 | 0.04 | 0.05 | 0.10 | 0.37 | 0.26 | 0.10 | 0.03 | 0.09 | 0.07 | 0.12 | 0.15 | 0.05 | 0.07 | 0.12 | 0.43 | 0.35 | 0.47 | 0.94 | 0.26 | 0.08 |
| -3.08 | SC | 0.07 | 0.02 | 0.05 | 0.06 | 0.02 | 0.02 | 0.04 | 0.05 | 0.01 | 0.16 | 0.22 | 0.26 | 0.08 | 0.02 | 0.15 | 0.05 | 0.14 | 0.15 | 0.11 | 0.11 | 0.09 | 0.19 | 0.18 | 0.23 | 0.55 | 0.18 | 0.04 |
| -3.08 | MGN | 0.05 | 0.02 | 0.05 | 0.01 | 0.03 | 0.03 | 0.03 | 0.03 | 0.00 | 0.08 | 0.19 | 0.06 | 0.05 | 0.01 | 0.05 | 0.09 | 0.05 | 0.05 | 0.02 | 0.04 | 0.05 | 0.08 | 0.03 | 0.08 | 0.25 | 0.07 | 0.02 |
| -3.08 | MRN | 0.05 | 0.02 | 0.05 | 0.01 | 0.03 | 0.03 | 0.03 | 0.03 | 0.00 | 0.08 | 0.14 | 0.06 | 0.04 | 0.01 | 0.07 | 0.09 | 0.08 | 0.15 | 0.04 | 0.08 | 0.08 | 0.19 | 0.17 | 0.12 | 0.47 | 0.14 | 0.04 |
| -3.08 | VTA | 0.03 | 0.01 | 0.01 | 0.04 | 0.06 | 0.01 | 0.02 | 0.05 | 0.34 | 0.02 | 0.07 | 0.08 | 0.06 | 0.03 | 0.09 | 0.09 | 0.07 | 0.08 | 0.04 | 0.08 | 0.06 | 0.43 | 0.02 | 0.12 | 0.69 | 0.16 | 0.06 |
| -3.08 | PAG | 0.12 | 0.04 | 0.04 | 0.00 | 0.03 | 0.04 | 0.04 | 0.05 | 0.00 | 0.24 | 0.38 | 0.42 | 0.12 | 0.04 | 0.12 | 0.2 | 0.2 | 0.19 | 0.07 | 0.12 | 0.15 | 0.82 | 0.48 | 0.32 | 1.14 | 0.35 | 0.10 |

**Supplementary Table 1**. **CRS aggravates α-syn propagation.** Raw data for Figure 2a

| Bregma | Regions | PBS A53T+NCRS | | | | | PBS A53T+CRS | | | | | | | PFF A53T+NCRS | | | | | | | PFF A53T+CRS | | | | | | | | |
| --- | --- | --- | --- | --- | --- | --- | --- | --- | --- | --- | --- | --- | --- | --- | --- | --- | --- | --- | --- | --- | --- | --- | --- | --- | --- | --- | --- | --- | --- |
|  |  | 1 | 2 | 3 | Mean | SEM | 1 | 2 | 3 | 4 | 5 | Mean | SEM | 1 | 2 | 3 | 4 | 5 | Mean | SEM | 1 | 2 | 3 | 4 | 5 | 6 | 7 | Mean | SEM |
| 2.1 | Cg | 0.11 | 0.06 | 0.12 | 0.10 | 0.02 | 0.20 | 0.53 | 0.25 | 0.37 | 0.19 | 0.31 | 0.06 | 0.31 | 0.40 | 0.27 | 0.22 | 0.30 | 0.30 | 0.03 | 0.47 | 0.53 | 0.41 | 0.30 | 0.51 | 0.44 | 0.50 | 0.45 | 0.03 |
| 2.1 | PrL | 0.17 | 0.12 | 0.11 | 0.14 | 0.02 | 0.21 | 0.42 | 0.32 | 0.30 | 0.15 | 0.28 | 0.05 | 0.28 | 0.33 | 0.26 | 0.16 | 0.22 | 0.25 | 0.03 | 0.55 | 0.54 | 0.56 | 0.47 | 0.65 | 0.49 | 0.50 | 0.54 | 0.02 |
| 2.1 | MO | 0.10 | 0.07 | 0.13 | 0.10 | 0.02 | 0.22 | 0.37 | 0.16 | 0.31 | 0.10 | 0.23 | 0.05 | 0.17 | 0.24 | 0.21 | 0.10 | 0.19 | 0.18 | 0.02 | 0.55 | 0.73 | 0.38 | 0.58 | 0.55 | 0.44 | 0.64 | 0.55 | 0.04 |
| 2.1 | M2 | 0.02 | 0.02 | 0.03 | 0.02 | 0.01 | 0.46 | 0.47 | 0.18 | 0.44 | 0.49 | 0.41 | 0.06 | 0.27 | 0.41 | 0.25 | 0.16 | 0.40 | 0.30 | 0.05 | 0.46 | 0.36 | 0.57 | 0.45 | 0.31 | 0.52 | 0.46 | 0.45 | 0.03 |
| 2.1 | M1 | 0.02 | 0.01 | 0.02 | 0.01 | 0.00 | 0.16 | 0.10 | 0.04 | 0.07 | 0.34 | 0.14 | 0.05 | 0.10 | 0.09 | 0.04 | 0.05 | 0.06 | 0.07 | 0.01 | 0.29 | 0.36 | 0.23 | 0.20 | 0.52 | 0.37 | 0.40 | 0.34 | 0.04 |
| 2.1 | AI | 0.02 | 0.07 | 0.03 | 0.04 | 0.02 | 0.31 | 0.20 | 0.05 | 0.05 | 0.17 | 0.16 | 0.05 | 0.06 | 0.10 | 0.13 | 0.06 | 0.10 | 0.09 | 0.01 | 0.45 | 0.51 | 0.39 | 0.11 | 0.20 | 0.14 | 0.08 | 0.27 | 0.07 |
| 2.1 | LO | 0.06 | 0.09 | 0.03 | 0.06 | 0.02 | 0.59 | 0.35 | 0.16 | 0.21 | 0.15 | 0.29 | 0.08 | 0.07 | 0.07 | 0.10 | 0.08 | 0.14 | 0.09 | 0.01 | 0.50 | 0.30 | 0.69 | 0.30 | 0.43 | 0.37 | 0.26 | 0.41 | 0.06 |
| 2.1 | VO | 0.02 | 0.03 | 0.04 | 0.03 | 0.01 | 0.30 | 0.17 | 0.11 | 0.22 | 0.15 | 0.19 | 0.03 | 0.07 | 0.15 | 0.05 | 0.11 | 0.27 | 0.13 | 0.04 | 0.47 | 0.39 | 0.54 | 0.67 | 0.74 | 0.39 | 0.57 | 0.54 | 0.05 |
| 2.1 | AO | 0.23 | 0.06 | 0.18 | 0.16 | 0.05 | 0.39 | 0.39 | 0.38 | 0.37 | 0.09 | 0.32 | 0.06 | 0.16 | 0.16 | 0.32 | 0.06 | 0.02 | 0.14 | 0.05 | 0.43 | 0.44 | 0.41 | 0.46 | 0.62 | 0.66 | 0.77 | 0.54 | 0.05 |
| 2.1 | TT | 0.09 | 0.25 | 0.20 | 0.18 | 0.05 | 0.30 | 0.16 | 0.29 | 0.17 | 0.14 | 0.21 | 0.03 | 0.25 | 0.08 | 0.32 | 0.07 | 0.02 | 0.15 | 0.06 | 0.32 | 0.36 | 0.28 | 0.50 | 0.69 | 0.55 | 0.66 | 0.48 | 0.06 |
| 2.1 | Pir | 0.11 | 0.27 | 0.13 | 0.17 | 0.05 | 0.38 | 0.33 | 0.17 | 0.23 | 0.05 | 0.23 | 0.06 | 0.13 | 0.27 | 0.27 | 0.08 | 0.02 | 0.15 | 0.05 | 0.30 | 0.31 | 0.28 | 0.56 | 0.78 | 0.64 | 0.79 | 0.52 | 0.09 |
| 0.98 | Cg | 0.15 | 0.09 | 0.07 | 0.10 | 0.02 | 0.09 | 0.21 | 0.14 | 0.24 | 0.21 | 0.18 | 0.03 | 0.16 | 0.28 | 0.37 | 0.36 | 0.41 | 0.31 | 0.04 | 0.56 | 0.44 | 0.67 | 0.59 | 0.57 | 0.59 | 0.69 | 0.59 | 0.03 |
| 0.98 | M2 | 0.06 | 0.07 | 0.07 | 0.07 | 0.00 | 0.23 | 0.31 | 0.33 | 0.27 | 0.40 | 0.31 | 0.03 | 0.36 | 0.32 | 0.48 | 0.46 | 0.37 | 0.40 | 0.03 | 0.53 | 0.44 | 0.42 | 0.61 | 0.54 | 0.61 | 0.45 | 0.51 | 0.03 |
| 0.98 | M1 | 0.03 | 0.02 | 0.03 | 0.03 | 0.00 | 0.37 | 0.33 | 0.31 | 0.25 | 0.51 | 0.35 | 0.04 | 0.25 | 0.39 | 0.25 | 0.26 | 0.20 | 0.27 | 0.03 | 0.38 | 0.28 | 0.48 | 0.25 | 0.14 | 0.13 | 0.50 | 0.31 | 0.06 |
| 0.98 | S1 | 0.02 | 0.02 | 0.04 | 0.03 | 0.01 | 0.12 | 0.09 | 0.14 | 0.11 | 0.18 | 0.13 | 0.01 | 0.10 | 0.11 | 0.19 | 0.18 | 0.10 | 0.14 | 0.02 | 0.18 | 0.13 | 0.24 | 0.14 | 0.03 | 0.18 | 0.20 | 0.16 | 0.03 |
| 0.98 | S2 | 0.09 | 0.05 | 0.06 | 0.07 | 0.01 | 0.15 | 0.04 | 0.09 | 0.06 | 0.12 | 0.09 | 0.02 | 0.04 | 0.04 | 0.22 | 0.07 | 0.06 | 0.09 | 0.03 | 0.27 | 0.19 | 0.35 | 0.01 | 0.03 | 0.05 | 0.05 | 0.14 | 0.05 |
| 0.98 | Ins | 0.04 | 0.15 | 0.10 | 0.09 | 0.03 | 0.18 | 0.24 | 0.24 | 0.18 | 0.25 | 0.22 | 0.02 | 0.11 | 0.20 | 0.33 | 0.30 | 0.40 | 0.27 | 0.05 | 0.42 | 0.37 | 0.47 | 0.48 | 0.58 | 0.38 | 0.51 | 0.46 | 0.03 |
| 0.98 | Pir | 0.23 | 0.30 | 0.15 | 0.23 | 0.04 | 0.13 | 0.17 | 0.18 | 0.09 | 0.18 | 0.15 | 0.02 | 0.20 | 0.06 | 0.48 | 0.04 | 0.01 | 0.16 | 0.09 | 0.20 | 0.12 | 0.28 | 0.55 | 0.42 | 0.31 | 0.50 | 0.34 | 0.06 |
| 0.98 | Cpu | 0.02 | 0.02 | 0.01 | 0.02 | 0.00 | 0.03 | 0.04 | 0.04 | 0.05 | 0.05 | 0.04 | 0.00 | 0.04 | 0.05 | 0.12 | 0.12 | 0.04 | 0.07 | 0.02 | 0.18 | 0.06 | 0.30 | 0.05 | 0.08 | 0.06 | 0.06 | 0.11 | 0.04 |
| 0.98 | Acb | 0.18 | 0.12 | 0.11 | 0.14 | 0.02 | 0.08 | 0.32 | 0.27 | 0.11 | 0.03 | 0.16 | 0.06 | 0.15 | 0.03 | 0.41 | 0.03 | 0.01 | 0.13 | 0.07 | 0.20 | 0.15 | 0.25 | 0.17 | 0.13 | 0.03 | 0.07 | 0.14 | 0.03 |
| 0.98 | Sep | 0.12 | 0.15 | 0.12 | 0.13 | 0.01 | 0.44 | 0.44 | 0.37 | 0.41 | 0.19 | 0.37 | 0.05 | 0.13 | 0.07 | 0.38 | 0.05 | 0.01 | 0.13 | 0.07 | 0.54 | 0.47 | 0.51 | 0.49 | 0.39 | 0.27 | 0.22 | 0.41 | 0.05 |
| -1.58 | RS | 0.11 | 0.06 | 0.19 | 0.12 | 0.04 | 0.09 | 0.16 | 0.18 | 0.12 | 0.11 | 0.13 | 0.02 | 0.31 | 0.19 | 0.43 | 0.05 | 0.12 | 0.22 | 0.07 | 0.36 | 0.27 | 0.40 | 0.26 | 0.32 | 0.31 | 0.19 | 0.30 | 0.03 |
| -1.58 | PtA | 0.06 | 0.04 | 0.11 | 0.07 | 0.02 | 0.19 | 0.19 | 0.38 | 0.29 | 0.19 | 0.25 | 0.04 | 0.11 | 0.05 | 0.10 | 0.05 | 0.40 | 0.14 | 0.07 | 0.26 | 0.43 | 0.12 | 0.64 | 0.66 | 0.69 | 0.41 | 0.46 | 0.08 |
| -1.58 | S1 | 0.08 | 0.04 | 0.08 | 0.07 | 0.02 | 0.40 | 0.38 | 0.42 | 0.28 | 0.09 | 0.31 | 0.06 | 0.13 | 0.06 | 0.16 | 0.08 | 0.09 | 0.10 | 0.02 | 0.35 | 0.39 | 0.43 | 0.54 | 0.61 | 0.48 | 0.40 | 0.46 | 0.03 |
| -1.58 | S2 | 0.08 | 0.01 | 0.09 | 0.06 | 0.03 | 0.30 | 0.49 | 0.29 | 0.17 | 0.25 | 0.30 | 0.05 | 0.26 | 0.16 | 0.29 | 0.06 | 0.15 | 0.19 | 0.04 | 0.40 | 0.34 | 0.40 | 0.45 | 0.40 | 0.34 | 0.32 | 0.38 | 0.02 |
| -1.58 | Ect | 0.17 | 0.07 | 0.06 | 0.10 | 0.04 | 0.28 | 0.36 | 0.12 | 0.12 | 0.46 | 0.27 | 0.07 | 0.31 | 0.29 | 0.19 | 0.10 | 0.19 | 0.22 | 0.04 | 0.58 | 0.23 | 0.71 | 0.49 | 0.37 | 0.18 | 0.29 | 0.41 | 0.07 |
| -1.58 | PRh | 0.08 | 0.08 | 0.14 | 0.10 | 0.02 | 0.21 | 0.13 | 0.12 | 0.09 | 0.36 | 0.18 | 0.05 | 0.23 | 0.09 | 0.22 | 0.12 | 0.15 | 0.16 | 0.03 | 0.35 | 0.16 | 0.73 | 0.24 | 0.36 | 0.18 | 0.28 | 0.33 | 0.07 |
| -1.58 | Pir | 0.15 | 0.13 | 0.08 | 0.12 | 0.02 | 0.10 | 0.13 | 0.19 | 0.08 | 0.16 | 0.13 | 0.02 | 0.44 | 0.23 | 0.45 | 0.31 | 0.18 | 0.32 | 0.05 | 0.35 | 0.38 | 0.23 | 0.30 | 0.46 | 0.54 | 0.47 | 0.39 | 0.04 |
| -1.58 | Ce | 0.08 | 0.12 | 0.15 | 0.12 | 0.02 | 0.18 | 0.21 | 0.14 | 0.17 | 0.08 | 0.16 | 0.02 | 0.39 | 0.14 | 0.03 | 0.02 | 0.00 | 0.12 | 0.07 | 0.54 | 0.57 | 0.41 | 0.45 | 0.31 | 0.59 | 0.48 | 0.48 | 0.04 |
| -1.58 | BLA | 0.12 | 0.11 | 0.16 | 0.13 | 0.01 | 0.31 | 0.32 | 0.31 | 0.30 | 0.16 | 0.28 | 0.03 | 0.26 | 0.26 | 0.39 | 0.26 | 0.21 | 0.28 | 0.03 | 0.62 | 0.64 | 0.70 | 0.52 | 0.52 | 0.41 | 0.52 | 0.56 | 0.04 |
| -1.58 | BMA | 0.15 | 0.07 | 0.08 | 0.10 | 0.02 | 0.14 | 0.16 | 0.16 | 0.13 | 0.14 | 0.15 | 0.01 | 0.34 | 0.11 | 0.61 | 0.16 | 0.12 | 0.27 | 0.10 | 0.56 | 0.49 | 0.56 | 0.52 | 0.33 | 0.30 | 0.42 | 0.45 | 0.04 |
| -1.58 | Me | 0.19 | 0.18 | 0.09 | 0.15 | 0.03 | 0.12 | 0.15 | 0.24 | 0.09 | 0.12 | 0.14 | 0.03 | 0.30 | 0.13 | 0.40 | 0.05 | 0.06 | 0.19 | 0.07 | 0.36 | 0.32 | 0.34 | 0.32 | 0.20 | 0.32 | 0.30 | 0.31 | 0.02 |
| -1.58 | Thal | 0.08 | 0.05 | 0.09 | 0.07 | 0.01 | 0.10 | 0.21 | 0.17 | 0.29 | 0.06 | 0.17 | 0.04 | 0.19 | 0.25 | 0.24 | 0.03 | 0.02 | 0.15 | 0.05 | 0.22 | 0.19 | 0.16 | 0.06 | 0.06 | 0.07 | 0.09 | 0.12 | 0.03 |
| -1.58 | Hth | 0.27 | 0.27 | 0.40 | 0.31 | 0.04 | 0.60 | 0.69 | 0.51 | 0.64 | 0.51 | 0.59 | 0.04 | 0.42 | 0.12 | 0.31 | 0.05 | 0.03 | 0.19 | 0.08 | 0.41 | 0.42 | 0.47 | 0.28 | 0.32 | 0.16 | 0.23 | 0.33 | 0.04 |
| -1.58 | MGP | 0.04 | 0.01 | 0.05 | 0.03 | 0.01 | 0.01 | 0.04 | 0.02 | 0.09 | 0.03 | 0.04 | 0.01 | 0.12 | 0.11 | 0.04 | 0.04 | 0.07 | 0.08 | 0.02 | 0.13 | 0.19 | 0.04 | 0.12 | 0.12 | 0.14 | 0.17 | 0.13 | 0.02 |
| -1.58 | Hipp | 0.09 | 0.06 | 0.05 | 0.07 | 0.01 | 0.13 | 0.07 | 0.07 | 0.05 | 0.02 | 0.07 | 0.02 | 0.11 | 0.11 | 0.13 | 0.07 | 0.11 | 0.10 | 0.01 | 0.15 | 0.15 | 0.16 | 0.08 | 0.12 | 0.10 | 0.06 | 0.12 | 0.01 |
| -3.08 | RS | 0.05 | 0.05 | 0.10 | 0.07 | 0.02 | 0.06 | 0.04 | 0.08 | 0.07 | 0.09 | 0.07 | 0.01 | 0.31 | 0.36 | 0.29 | 0.05 | 0.12 | 0.23 | 0.06 | 0.26 | 0.37 | 0.35 | 0.45 | 0.38 | 0.48 | 0.31 | 0.37 | 0.03 |
| -3.08 | V | 0.11 | 0.11 | 0.08 | 0.10 | 0.01 | 0.14 | 0.08 | 0.17 | 0.19 | 0.04 | 0.12 | 0.03 | 0.34 | 0.24 | 0.46 | 0.27 | 0.20 | 0.30 | 0.05 | 0.43 | 0.12 | 0.73 | 0.70 | 0.43 | 0.36 | 0.38 | 0.45 | 0.08 |
| -3.08 | Au | 0.34 | 0.33 | 0.16 | 0.27 | 0.06 | 0.46 | 0.26 | 0.42 | 0.21 | 0.03 | 0.28 | 0.08 | 0.39 | 0.31 | 0.34 | 0.18 | 0.29 | 0.30 | 0.03 | 0.31 | 0.48 | 0.43 | 0.41 | 0.59 | 0.44 | 0.34 | 0.43 | 0.03 |
| -3.08 | TeA | 0.19 | 0.19 | 0.17 | 0.18 | 0.01 | 0.35 | 0.33 | 0.36 | 0.16 | 0.15 | 0.27 | 0.05 | 0.22 | 0.25 | 0.15 | 0.16 | 0.25 | 0.21 | 0.02 | 0.55 | 0.51 | 0.59 | 0.44 | 0.33 | 0.21 | 0.31 | 0.42 | 0.05 |
| -3.08 | Ect | 0.24 | 0.22 | 0.11 | 0.19 | 0.04 | 0.29 | 0.41 | 0.31 | 0.31 | 0.13 | 0.29 | 0.04 | 0.23 | 0.34 | 0.29 | 0.30 | 0.29 | 0.29 | 0.02 | 0.48 | 0.51 | 0.45 | 0.26 | 0.31 | 0.39 | 0.38 | 0.40 | 0.03 |
| -3.08 | PRh | 0.07 | 0.04 | 0.09 | 0.07 | 0.01 | 0.14 | 0.34 | 0.18 | 0.23 | 0.27 | 0.24 | 0.03 | 0.33 | 0.27 | 0.21 | 0.12 | 0.15 | 0.22 | 0.04 | 0.37 | 0.35 | 0.38 | 0.49 | 0.31 | 0.40 | 0.23 | 0.36 | 0.03 |
| -3.08 | Ent | 0.14 | 0.18 | 0.18 | 0.17 | 0.01 | 0.27 | 0.18 | 0.21 | 0.25 | 0.35 | 0.25 | 0.03 | 0.27 | 0.34 | 0.25 | 0.31 | 0.38 | 0.31 | 0.02 | 0.50 | 0.56 | 0.44 | 0.44 | 0.43 | 0.43 | 0.63 | 0.49 | 0.03 |
| -3.08 | Sub | 0.00 | 0.01 | 0.02 | 0.01 | 0.01 | 0.06 | 0.02 | 0.03 | 0.05 | 0.05 | 0.04 | 0.01 | 0.11 | 0.22 | 0.23 | 0.02 | 0.04 | 0.12 | 0.04 | 0.14 | 0.12 | 0.16 | 0.05 | 0.72 | 0.17 | 0.15 | 0.22 | 0.09 |
| -3.08 | Hipp | 0.11 | 0.09 | 0.09 | 0.09 | 0.01 | 0.07 | 0.09 | 0.07 | 0.09 | 0.02 | 0.07 | 0.01 | 0.18 | 0.26 | 0.16 | 0.06 | 0.01 | 0.14 | 0.04 | 0.27 | 0.27 | 0.28 | 0.12 | 0.14 | 0.10 | 0.11 | 0.18 | 0.03 |
| -3.08 | SN | 0.04 | 0.03 | 0.03 | 0.03 | 0.00 | 0.11 | 0.10 | 0.08 | 0.06 | 0.12 | 0.09 | 0.01 | 0.27 | 0.46 | 0.03 | 0.16 | 0.12 | 0.21 | 0.07 | 0.27 | 0.33 | 0.23 | 0.09 | 0.27 | 0.37 | 0.31 | 0.27 | 0.03 |
| -3.08 | SC | 0.25 | 0.16 | 0.24 | 0.22 | 0.03 | 0.38 | 0.30 | 0.13 | 0.28 | 0.10 | 0.24 | 0.05 | 0.23 | 0.60 | 0.25 | 0.25 | 0.16 | 0.30 | 0.08 | 0.28 | 0.49 | 0.28 | 0.15 | 0.38 | 0.30 | 0.24 | 0.30 | 0.04 |
| -3.08 | MGN | 0.04 | 0.07 | 0.05 | 0.05 | 0.01 | 0.08 | 0.15 | 0.10 | 0.34 | 0.03 | 0.14 | 0.05 | 0.31 | 0.22 | 0.04 | 0.09 | 0.18 | 0.17 | 0.05 | 0.23 | 0.22 | 0.24 | 0.35 | 0.18 | 0.19 | 0.44 | 0.27 | 0.04 |
| -3.08 | MRN | 0.12 | 0.08 | 0.10 | 0.10 | 0.01 | 0.13 | 0.32 | 0.11 | 0.32 | 0.08 | 0.19 | 0.05 | 0.20 | 0.41 | 0.09 | 0.15 | 0.13 | 0.19 | 0.06 | 0.22 | 0.30 | 0.14 | 0.35 | 0.32 | 0.33 | 0.24 | 0.27 | 0.03 |
| -3.08 | VTA | 0.08 | 0.05 | 0.06 | 0.06 | 0.01 | 0.15 | 0.28 | 0.11 | 0.14 | 0.07 | 0.15 | 0.03 | 0.18 | 0.12 | 0.04 | 0.14 | 0.07 | 0.11 | 0.03 | 0.17 | 0.21 | 0.12 | 0.28 | 0.29 | 0.18 | 0.16 | 0.20 | 0.02 |
| -3.08 | PAG | 0.33 | 0.21 | 0.20 | 0.25 | 0.04 | 0.47 | 0.54 | 0.47 | 0.60 | 0.09 | 0.43 | 0.09 | 0.35 | 0.28 | 0.34 | 0.27 | 0.39 | 0.33 | 0.02 | 0.63 | 0.86 | 0.40 | 0.31 | 0.39 | 0.56 | 0.68 | 0.55 | 0.07 |

**Supplementary Table 2. CRS leads to increased α-syn propagation associated with c-Fos induction.** Raw data for Figure 3a

| Bregma | Regions | PFF A53T + PBS | | | | | | PFF A53T + MPEP | | | | | |
| --- | --- | --- | --- | --- | --- | --- | --- | --- | --- | --- | --- | --- | --- |
|  |  | 1 | 2 | 3 | 4 | Mean | SEM | 1 | 2 | 3 | 4 | Mean | SEM |
| 2.1 | Cg | 0.64 | 0.65 | 1.15 | 0.93 | 0.84 | 0.12 | 0.32 | 0.55 | 0.23 | 0.54 | 0.41 | 0.08 |
| 2.1 | PrL | 0.40 | 0.31 | 0.73 | 0.41 | 0.46 | 0.09 | 0.44 | 0.22 | 0.44 | 0.32 | 0.36 | 0.05 |
| 2.1 | MO | 0.29 | 0.11 | 0.15 | 0.28 | 0.21 | 0.05 | 0.41 | 0.18 | 0.46 | 0.41 | 0.37 | 0.06 |
| 2.1 | M2 | 1.04 | 1.22 | 0.76 | 1.21 | 1.06 | 0.11 | 0.15 | 0.36 | 0.17 | 0.56 | 0.31 | 0.10 |
| 2.1 | M1 | 0.72 | 0.46 | 0.33 | 0.66 | 0.54 | 0.09 | 0.06 | 0.04 | 0.01 | 0.18 | 0.07 | 0.04 |
| 2.1 | AI | 0.68 | 0.42 | 0.47 | 0.87 | 0.61 | 0.10 | 0.02 | 0.11 | 0.01 | 0.10 | 0.06 | 0.03 |
| 2.1 | LO | 0.54 | 0.29 | 0.13 | 0.29 | 0.31 | 0.08 | 0.11 | 0.05 | 0.00 | 0.09 | 0.06 | 0.02 |
| 2.1 | VO | 0.42 | 0.34 | 0.07 | 0.35 | 0.30 | 0.08 | 0.43 | 0.27 | 0.09 | 0.10 | 0.22 | 0.08 |
| 2.1 | AO | 0.02 | 0.00 | 0.17 | 0.01 | 0.05 | 0.04 | 0.01 | 0.01 | 0.08 | 0.00 | 0.03 | 0.02 |
| 2.1 | TT | 0.01 | 0.01 | 0.10 | 0.01 | 0.03 | 0.02 | 0.01 | 0.05 | 0.17 | 0.01 | 0.06 | 0.04 |
| 2.1 | Pir | 0.01 | 0.02 | 0.08 | 0.01 | 0.03 | 0.02 | 0.00 | 0.01 | 0.02 | 0.00 | 0.01 | 0.00 |
| 0.98 | Cg | 0.75 | 1.04 | 0.62 | 0.68 | 0.77 | 0.09 | 0.66 | 0.20 | 0.18 | 0.65 | 0.42 | 0.13 |
| 0.98 | M2 | 1.05 | 1.09 | 0.68 | 1.04 | 0.97 | 0.10 | 0.29 | 0.45 | 0.09 | 0.62 | 0.36 | 0.11 |
| 0.98 | M1 | 0.52 | 0.62 | 0.52 | 0.33 | 0.50 | 0.06 | 0.22 | 0.32 | 0.02 | 0.19 | 0.19 | 0.06 |
| 0.98 | S1 | 0.12 | 0.24 | 0.01 | 0.05 | 0.11 | 0.05 | 0.07 | 0.34 | 0.00 | 0.13 | 0.14 | 0.07 |
| 0.98 | S2 | 0.18 | 0.37 | 0.09 | 0.26 | 0.23 | 0.06 | 0.06 | 0.16 | 0.02 | 0.41 | 0.16 | 0.09 |
| 0.98 | Ins | 0.24 | 0.47 | 0.38 | 0.26 | 0.34 | 0.05 | 0.12 | 0.12 | 0.50 | 0.21 | 0.24 | 0.09 |
| 0.98 | Pir | 0.03 | 0.01 | 0.17 | 0.07 | 0.07 | 0.04 | 0.01 | 0.01 | 0.18 | 0.00 | 0.05 | 0.04 |
| 0.98 | CPu | 0.15 | 0.07 | 0.32 | 0.24 | 0.20 | 0.05 | 0.10 | 0.03 | 0.04 | 0.04 | 0.05 | 0.02 |
| 0.98 | Acb | 0.00 | 0.01 | 0.16 | 0.03 | 0.05 | 0.04 | 0.03 | 0.01 | 0.07 | 0.00 | 0.03 | 0.02 |
| 0.98 | Sep | 0.04 | 0.05 | 0.27 | 0.01 | 0.09 | 0.06 | 0.17 | 0.00 | 0.35 | 0.01 | 0.13 | 0.08 |
| -1.58 | RS | 0.10 | 0.09 | 0.13 | 0.17 | 0.12 | 0.02 | 0.04 | 0.11 | 0.02 | 0.11 | 0.07 | 0.02 |
| -1.58 | PtA | 0.26 | 0.15 | 0.23 | 0.20 | 0.21 | 0.02 | 0.07 | 0.35 | 0.02 | 0.30 | 0.19 | 0.08 |
| -1.58 | S1 | 0.08 | 0.05 | 0.02 | 0.10 | 0.06 | 0.02 | 0.05 | 0.12 | 0.01 | 0.05 | 0.06 | 0.02 |
| -1.58 | S2 | 0.19 | 0.14 | 0.08 | 0.14 | 0.14 | 0.02 | 0.08 | 0.24 | 0.03 | 0.22 | 0.14 | 0.05 |
| -1.58 | Ect | 0.39 | 0.22 | 0.19 | 0.33 | 0.28 | 0.05 | 0.07 | 0.31 | 0.11 | 0.25 | 0.19 | 0.06 |
| -1.58 | PRh | 0.36 | 0.27 | 0.15 | 0.62 | 0.35 | 0.10 | 0.13 | 0.52 | 0.32 | 0.23 | 0.30 | 0.08 |
| -1.58 | Pir | 0.13 | 0.07 | 0.16 | 0.23 | 0.15 | 0.03 | 0.07 | 0.05 | 0.31 | 0.02 | 0.11 | 0.07 |
| -1.58 | Ce | 0.44 | 0.21 | 0.49 | 0.30 | 0.36 | 0.06 | 0.10 | 0.27 | 0.48 | 0.50 | 0.34 | 0.09 |
| -1.58 | BLA | 0.80 | 0.34 | 0.56 | 0.41 | 0.53 | 0.10 | 0.12 | 0.53 | 0.30 | 0.51 | 0.37 | 0.10 |
| -1.58 | Thal | 0.09 | 0.10 | 0.23 | 0.15 | 0.14 | 0.03 | 0.04 | 0.06 | 0.04 | 0.07 | 0.05 | 0.01 |
| -1.58 | Hippo | 0.01 | 0.00 | 0.02 | 0.00 | 0.01 | 0.00 | 0.02 | 0.00 | 0.00 | 0.01 | 0.01 | 0.00 |
| -3.08 | RS | 0.10 | 0.13 | 0.10 | 0.05 | 0.10 | 0.02 | 0.03 | 0.11 | 0.02 | 0.06 | 0.06 | 0.02 |
| -3.08 | V | 0.14 | 0.19 | 0.04 | 0.07 | 0.11 | 0.03 | 0.17 | 0.23 | 0.01 | 0.08 | 0.12 | 0.05 |
| -3.08 | Au | 0.07 | 0.15 | 0.08 | 0.19 | 0.12 | 0.03 | 0.09 | 0.23 | 0.01 | 0.10 | 0.11 | 0.05 |
| -3.08 | TeA | 0.10 | 0.29 | 0.27 | 0.10 | 0.19 | 0.05 | 0.20 | 0.35 | 0.05 | 0.09 | 0.17 | 0.07 |
| -3.08 | Ect | 0.15 | 0.47 | 0.21 | 0.47 | 0.33 | 0.08 | 0.36 | 0.36 | 0.16 | 0.16 | 0.26 | 0.06 |
| -3.08 | PRh | 0.19 | 0.31 | 0.26 | 0.40 | 0.29 | 0.04 | 0.19 | 0.37 | 0.15 | 0.18 | 0.22 | 0.05 |
| -3.08 | Ent | 0.08 | 0.09 | 0.64 | 0.09 | 0.23 | 0.14 | 0.33 | 0.22 | 0.43 | 0.08 | 0.27 | 0.08 |
| -3.08 | Sub | 0.02 | 0.01 | 0.15 | 0.01 | 0.05 | 0.03 | 0.03 | 0.01 | 0.02 | 0.01 | 0.02 | 0.00 |
| -3.08 | Hippo | 0.01 | 0.01 | 0.02 | 0.01 | 0.01 | 0.00 | 0.05 | 0.00 | 0.01 | 0.01 | 0.02 | 0.01 |
| -3.08 | SN | 0.29 | 0.73 | 0.37 | 0.63 | 0.51 | 0.10 | 0.13 | 0.33 | 0.04 | 0.28 | 0.20 | 0.07 |
| -3.08 | SC | 0.10 | 0.13 | 0.26 | 0.13 | 0.16 | 0.04 | 0.06 | 0.09 | 0.02 | 0.06 | 0.06 | 0.01 |
| -3.08 | MGN | 0.03 | 0.08 | 0.22 | 0.08 | 0.10 | 0.04 | 0.08 | 0.04 | 0.12 | 0.01 | 0.06 | 0.02 |
| -3.08 | MRN | 0.08 | 0.13 | 0.16 | 0.24 | 0.15 | 0.03 | 0.04 | 0.07 | 0.05 | 0.08 | 0.06 | 0.01 |
| -3.08 | VTA | 0.06 | 0.12 | 0.45 | 0.14 | 0.19 | 0.09 | 0.06 | 0.06 | 0.17 | 0.14 | 0.11 | 0.03 |
| -3.08 | PAG | 0.31 | 0.40 | 0.79 | 0.44 | 0.49 | 0.11 | 0.42 | 0.23 | 0.28 | 0.18 | 0.28 | 0.05 |

**Supplementary Table 3**. **MPEP treatment reverses the effects of PFF injection on α-syn pathology.** Raw data for Figure 5c.

| Bregma | Regions | PFF A53T + PBS | | | | | | | PFF A53T + MPEP | | | | | |
| --- | --- | --- | --- | --- | --- | --- | --- | --- | --- | --- | --- | --- | --- | --- |
|  |  | 1 | 2 | 3 | 4 | 5 | Mean | SEM | 1 | 2 | 3 | 4 | Mean | SEM |
| 2.1 | Cg | 0.60 | 1.01 | 0.87 | 1.00 | 1.04 | 0.90 | 0.08 | 0.65 | 0.72 | 0.56 | 0.43 | 0.59 | 0.06 |
| 2.1 | PrL | 0.94 | 1.30 | 0.98 | 1.01 | 0.97 | 1.04 | 0.07 | 0.53 | 0.78 | 0.51 | 0.52 | 0.59 | 0.06 |
| 2.1 | MO | 1.17 | 1.10 | 0.87 | 1.28 | 1.04 | 1.09 | 0.07 | 0.32 | 0.82 | 0.50 | 0.86 | 0.63 | 0.13 |
| 2.1 | M2 | 0.50 | 0.62 | 0.64 | 0.52 | 0.34 | 0.52 | 0.05 | 0.28 | 0.31 | 0.38 | 0.22 | 0.30 | 0.03 |
| 2.1 | M1 | 0.02 | 0.10 | 0.01 | 0.02 | 0.04 | 0.04 | 0.02 | 0.01 | 0.05 | 0.06 | 0.01 | 0.03 | 0.01 |
| 2.1 | AI | 0.23 | 0.40 | 0.29 | 0.15 | 0.39 | 0.29 | 0.05 | 0.12 | 0.15 | 0.39 | 0.30 | 0.24 | 0.06 |
| 2.1 | LO | 0.59 | 0.87 | 0.73 | 0.51 | 1.06 | 0.75 | 0.10 | 0.36 | 0.14 | 0.50 | 0.55 | 0.39 | 0.09 |
| 2.1 | VO | 1.33 | 1.48 | 0.78 | 1.15 | 1.21 | 1.19 | 0.12 | 0.15 | 0.80 | 0.58 | 0.60 | 0.53 | 0.14 |
| 2.1 | AO | 0.91 | 1.25 | 1.31 | 1.54 | 1.31 | 1.27 | 0.10 | 0.57 | 1.11 | 0.49 | 1.18 | 0.84 | 0.18 |
| 2.1 | TT | 1.01 | 1.37 | 1.11 | 1.32 | 1.25 | 1.21 | 0.07 | 0.64 | 1.15 | 0.47 | 1.09 | 0.84 | 0.17 |
| 2.1 | Pir | 1.13 | 1.55 | 1.27 | 1.57 | 1.26 | 1.36 | 0.09 | 0.76 | 1.22 | 1.39 | 0.95 | 1.08 | 0.14 |
| 0.98 | Cg | 1.19 | 1.55 | 1.57 | 1.38 | 1.15 | 1.37 | 0.09 | 0.49 | 0.97 | 0.77 | 0.79 | 0.75 | 0.10 |
| 0.98 | M2 | 1.21 | 1.08 | 1.23 | 0.90 | 0.95 | 1.07 | 0.07 | 0.50 | 0.94 | 0.72 | 0.55 | 0.68 | 0.10 |
| 0.98 | M1 | 0.50 | 0.28 | 0.25 | 0.19 | 0.13 | 0.27 | 0.06 | 0.20 | 0.27 | 0.21 | 0.03 | 0.18 | 0.05 |
| 0.98 | S1 | 0.03 | 0.05 | 0.02 | 0.02 | 0.01 | 0.03 | 0.01 | 0.05 | 0.03 | 0.08 | 0.01 | 0.04 | 0.01 |
| 0.98 | S2 | 0.01 | 0.07 | 0.01 | 0.01 | 0.01 | 0.02 | 0.01 | 0.03 | 0.04 | 0.06 | 0.04 | 0.04 | 0.01 |
| 0.98 | Ins | 0.17 | 0.36 | 0.26 | 0.42 | 0.20 | 0.28 | 0.05 | 0.14 | 0.33 | 0.31 | 0.30 | 0.27 | 0.04 |
| 0.98 | Pir | 1.10 | 0.85 | 0.62 | 1.00 | 0.65 | 0.84 | 0.09 | 0.39 | 0.67 | 0.31 | 0.59 | 0.49 | 0.08 |
| 0.98 | CPu | 0.09 | 0.15 | 0.12 | 0.12 | 0.12 | 0.12 | 0.01 | 0.05 | 0.10 | 0.08 | 0.04 | 0.07 | 0.01 |
| 0.98 | Acb | 0.34 | 0.27 | 0.07 | 0.13 | 0.14 | 0.19 | 0.05 | 0.06 | 0.13 | 0.18 | 0.15 | 0.13 | 0.02 |
| 0.98 | Sep | 0.85 | 0.77 | 0.55 | 0.44 | 0.68 | 0.66 | 0.07 | 0.22 | 0.39 | 0.15 | 0.51 | 0.32 | 0.08 |
| -1.58 | RS | 0.51 | 0.64 | 0.62 | 0.38 | 0.37 | 0.51 | 0.06 | 0.37 | 0.53 | 0.45 | 0.43 | 0.44 | 0.03 |
| -1.58 | PtA | 1.29 | 1.31 | 1.38 | 0.83 | 1.14 | 1.19 | 0.10 | 0.76 | 0.88 | 0.81 | 0.67 | 0.78 | 0.04 |
| -1.58 | S1 | 1.08 | 1.21 | 0.96 | 0.79 | 0.89 | 0.99 | 0.07 | 0.95 | 1.02 | 0.98 | 0.57 | 0.88 | 0.10 |
| -1.58 | S2 | 0.90 | 0.79 | 0.68 | 0.64 | 0.65 | 0.73 | 0.05 | 1.00 | 0.88 | 0.71 | 0.62 | 0.80 | 0.09 |
| -1.58 | Ect | 0.98 | 0.74 | 0.35 | 0.57 | 0.67 | 0.66 | 0.10 | 0.86 | 0.69 | 0.62 | 0.49 | 0.66 | 0.08 |
| -1.58 | PRh | 0.47 | 0.73 | 0.35 | 0.55 | 0.42 | 0.50 | 0.06 | 0.30 | 0.39 | 0.46 | 0.44 | 0.40 | 0.04 |
| -1.58 | Pir | 0.61 | 0.92 | 1.07 | 0.94 | 0.66 | 0.84 | 0.09 | 0.62 | 0.79 | 0.37 | 0.59 | 0.59 | 0.08 |
| -1.58 | Ce | 0.19 | 0.26 | 0.06 | 0.16 | 0.22 | 0.18 | 0.03 | 0.07 | 0.22 | 0.16 | 0.26 | 0.18 | 0.04 |
| -1.58 | BLA | 0.41 | 0.49 | 0.28 | 0.44 | 0.47 | 0.42 | 0.04 | 0.30 | 0.42 | 0.39 | 0.32 | 0.36 | 0.03 |
| -1.58 | BMA | 0.32 | 0.56 | 0.60 | 0.46 | 0.61 | 0.51 | 0.06 | 0.42 | 0.54 | 0.22 | 0.65 | 0.46 | 0.09 |
| -1.58 | Me | 0.32 | 0.40 | 0.49 | 0.61 | 0.84 | 0.53 | 0.09 | 0.56 | 0.77 | 0.20 | 0.53 | 0.51 | 0.12 |
| -1.58 | Thal | 0.12 | 0.12 | 0.14 | 0.17 | 0.11 | 0.13 | 0.01 | 0.09 | 0.07 | 0.11 | 0.08 | 0.09 | 0.01 |
| -1.58 | Hth | 0.56 | 0.63 | 0.32 | 0.47 | 0.70 | 0.54 | 0.07 | 0.33 | 0.47 | 0.33 | 0.55 | 0.42 | 0.05 |
| -1.58 | Hipp | 0.15 | 0.24 | 0.20 | 0.12 | 0.14 | 0.17 | 0.02 | 0.11 | 0.14 | 0.06 | 0.15 | 0.12 | 0.02 |
| -3.08 | RS | 0.90 | 0.76 | 0.96 | 0.62 | 0.78 | 0.80 | 0.06 | 0.35 | 0.73 | 0.26 | 0.62 | 0.49 | 0.11 |
| -3.08 | V | 2.00 | 1.67 | 1.52 | 1.55 | 1.25 | 1.60 | 0.12 | 1.01 | 1.14 | 0.78 | 0.99 | 0.98 | 0.07 |
| -3.08 | Au | 0.83 | 1.18 | 0.48 | 0.69 | 0.70 | 0.78 | 0.11 | 0.21 | 0.83 | 0.22 | 0.56 | 0.45 | 0.15 |
| -3.08 | TeA | 0.87 | 0.66 | 0.41 | 0.63 | 0.83 | 0.68 | 0.08 | 0.16 | 0.48 | 0.37 | 0.75 | 0.44 | 0.12 |
| -3.08 | Ect | 0.52 | 0.62 | 0.79 | 0.56 | 0.60 | 0.62 | 0.05 | 0.60 | 0.61 | 0.31 | 0.64 | 0.54 | 0.08 |
| -3.08 | PRh | 0.99 | 0.62 | 0.81 | 0.46 | 0.60 | 0.70 | 0.09 | 0.38 | 0.52 | 0.28 | 0.62 | 0.45 | 0.08 |
| -3.08 | Ent | 0.88 | 0.53 | 0.51 | 0.51 | 0.47 | 0.58 | 0.07 | 0.28 | 0.50 | 0.29 | 0.60 | 0.42 | 0.08 |
| -3.08 | Sub | 0.10 | 0.14 | 0.03 | 0.10 | 0.06 | 0.09 | 0.02 | 0.04 | 0.03 | 0.04 | 0.04 | 0.04 | 0.00 |
| -3.08 | Hipp | 0.24 | 0.28 | 0.20 | 0.22 | 0.21 | 0.23 | 0.01 | 0.07 | 0.21 | 0.06 | 0.19 | 0.13 | 0.04 |
| -3.08 | SN | 0.19 | 0.01 | 0.01 | 0.02 | 0.03 | 0.05 | 0.03 | 0.02 | 0.01 | 0.07 | 0.06 | 0.04 | 0.01 |
| -3.08 | SC | 0.30 | 0.16 | 0.20 | 0.27 | 0.16 | 0.22 | 0.03 | 0.12 | 0.27 | 0.24 | 0.22 | 0.21 | 0.03 |
| -3.08 | MGN | 0.10 | 0.17 | 0.18 | 0.29 | 0.10 | 0.17 | 0.03 | 0.16 | 0.15 | 0.20 | 0.09 | 0.15 | 0.02 |
| -3.08 | MRN | 0.09 | 0.06 | 0.06 | 0.09 | 0.08 | 0.08 | 0.01 | 0.09 | 0.10 | 0.05 | 0.08 | 0.08 | 0.01 |
| -3.08 | VTA | 0.16 | 0.17 | 0.17 | 0.12 | 0.34 | 0.19 | 0.04 | 0.28 | 0.19 | 0.18 | 0.13 | 0.20 | 0.03 |
| -3.08 | PAG | 0.22 | 0.38 | 0.31 | 0.17 | 0.35 | 0.29 | 0.04 | 0.26 | 0.24 | 0.32 | 0.38 | 0.30 | 0.03 |

**Supplementary Table 4. MPEP treatment inhibits neuronal activity as measured by c-Fos induction induced by PFF injection.** Raw data for Figure 5d.
